# Toward a Systematics for the Lowest Excited States of Heteroaromatics Enabled via Cyclic π‑Conjugated Carbenes and Heteroelement Analogues

**DOI:** 10.1021/acs.joc.5c00578

**Published:** 2025-07-08

**Authors:** Nathalie Proos Vedin, Josene M. Toldo, Sílvia Escayola, Slavko Radenković, Miquel Solà, Henrik Ottosson

**Affiliations:** † Department of Chemistry - Ångström, 8097 Uppsala University, 751 20 Uppsala, Sweden; ‡ 27098 Université Claude Bernard Lyon 1, ENS de Lyon, CNRS, Laboratoire de Chimie, UMR 5182, 69342 Lyon Cedex 07, France; § Institut de Química Computacional i Catàlisi and Departament de Química, 16738 Universitat de Girona, C/Maria Aurèlia Capmany, 69, 17003 Girona, Catalonia, Spain; ∥ Donostia International Physics Center (DIPC), 20018 Donostia, Euskadi, Spain; ⊥ Institute for Theoretical Chemistry, University of Stuttgart, Pfaffenwaldring 55, 70569 Stuttgart, Germany; # Faculty of Science, 127740 University of Kragujevac, P.O. Box 60, 34000 Kragujevac, Serbia

## Abstract

The photochemistry of heteroaromatic compounds depends on the character of their lowest electronically excited states, which are of either *n*,π* or π,π* type. For species with 4*n* + 2 π-electrons, the latter type of states can be antiaromatic to various extents according to Baird’s rule and, thus, highly reactive. The *n*,π* type of states, on the other hand, will have an odd number of π-electrons leading to an unclear character, spanning from aromatic to antiaromatic. Six-membered ring (6-MR) heteroaromatics with in-plane lone-pair orbitals (*n*_σ_, herein *n*) have either *n*,π* or π,π* states as their lowest vertically excited states, but regular five-membered ring (5-MR) heteroaromatics with one or two N, O, and/or S atoms never have *n*,π* states as these states. However, this is different for cyclic π-conjugated (potentially aromatic) 5-MR carbenes that have the *n* orbitals at the divalent C atom. Also, 3-MR species have *n*,π* states as their lowest vertically excited states. Herein, we reveal which factors determine which type of vertical excited state is the lowest in energy for various heteroaromatics. The important factors are (i) the electronegativity of the heteroatom(s), (ii) the valence angle at the heteroatom impacting the lone-pair orbital energy, (iii) the number of π-orbitals and π-electrons, (iv) the degree of (anti)­aromatic character of the *n*,π* state, (v) the electronegativity of atoms adjacent to the heteroatom, and (vi) the spatial extent of the *n* orbital affecting the intraorbital electron repulsion. Our findings point toward the development of a rational systematics for prediction of which heteroaromatics have *n*,π* as the first vertical excited states and which ones have π,π* states as these.

## Graphical abstract

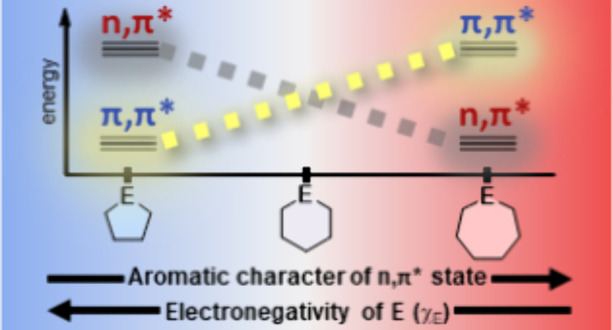

## Introduction

Heteroaromatic rings are found in various fields of chemistry and related sciences. The inclusion of heteroatoms within the rings modulates their electronic, steric, and biological properties, and their reactivity can be fine-tuned by the specific choice and position of the heteroatoms.^1^ They are common constituents of many drugs, bioactive molecules, agrochemicals, and organic electronics materials, besides being versatile building blocks in the synthesis of complex organic molecules.^2–10^ For many applications, the characters of their lowest electronically excited states are crucial.

Heteroaromatic molecules with in-plane lone-pairs (*n*_σ_, here labeled *n*), such as pyridine and thiophene, have *n*,π* excited states in addition to π,π* states, where transitions to the first are normally forbidden (dark) while those to the latter are allowed (bright). Despite this, conversions between the two types of states can occur in the excited state and impact on the further photochemistry and/or decay to the ground state (S_0_).^11,12^ We recently revealed that the *n*,π* states can have either aromatic, antiaromatic, or nonaromatic character.^13^ On the other hand, when excited to their lowest triplet excited state (T_1_) of π,π* type, the character changes more drastically as these states are antiaromatic according to Baird’s rule.^14–21^ This is also often the case in the lowest singlet π,π* states.^1,22–28^ When the *n*,π* state is of lower energy, as in pyrazine,^11,12^ the conversion from π,π* to *n*,π* is a route that alleviates excited state antiaromaticity.

In the present work, we explore the lowest few excited states of π-conjugated cyclic carbenes and some heteroelement analogues within the framework of heteroaromatics. As will be seen, this allows us to decipher what factors determine the character of the lowest excited states of heteroaromatics (*n*,π* or π,π*) with in-plane lone-pairs. The carbenes investigated have closed-shell singlet ground states (S_0_), which (formally) are Hückel-aromatic, and they have in-plane electron lone-pairs at the divalent carbon atom (or heteroatom). For such 6-, 5-, and 3-membered ring (-MR) carbenes and their isoelectronic heteroatom analogues (Figure 1), we now explored to what extent their *n*,π* states have aromatic or antiaromatic character.

**Figure 1:**
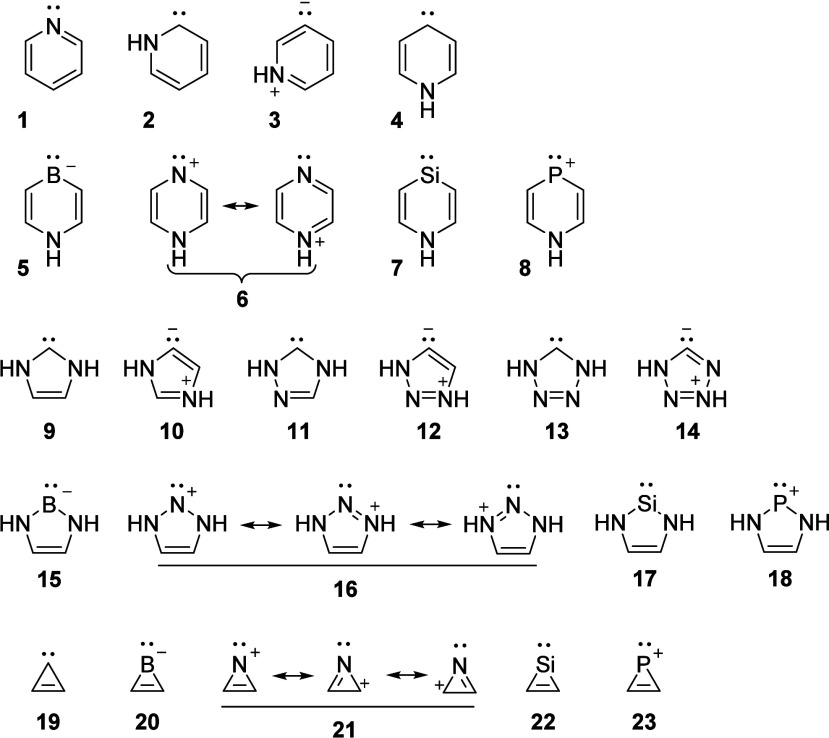
Pyridine and the twenty-two carbenes and (valence) isoelectronic heteroatom analogues investigated herein.

In our recent work, we found that 5-MR heteroaromatics with one or two N, O, and/or S atoms (e.g., thiophene and oxazole) never have *n*,π* states as their lowest valence excited states.^13^ Conversely, 6-MRs with one or two heteroatoms have *n*,π* states as their T_1_ and S_1_ states when the heteroatoms have low electronegativity and/or when the molecule has a high symmetry (∼*D*_2h_). But why do 5-MR heteroaromatics not have *n*,π* states as their lowest excited states? Our earlier investigation pointed to two factors: (i) the higher electronegativities of the heteroatom compared to C, and (ii) the more acute valence angles in the 5-MR than in the 6-MR compounds lead to a large relative lowering of the *n* orbital energy compared to the π and π* orbitals. As a result, the excitation energies for the *n*,π* transition (*E*(*n*,π*)) of 5-MR heteroaromatics are higher than those of 6-MRs. We now argue that the *n* orbitals of (formally) aromatic carbenes are of sufficiently high energies so that these species may have *n*,π* states as their T_1_ and S_1_ states. Indeed, dialkyl substituted carbenes normally have triplet ground states,^29^ which should be analogous to the triplet *n*,π* states studied herein (Figure 2A).

**Figure 2:**
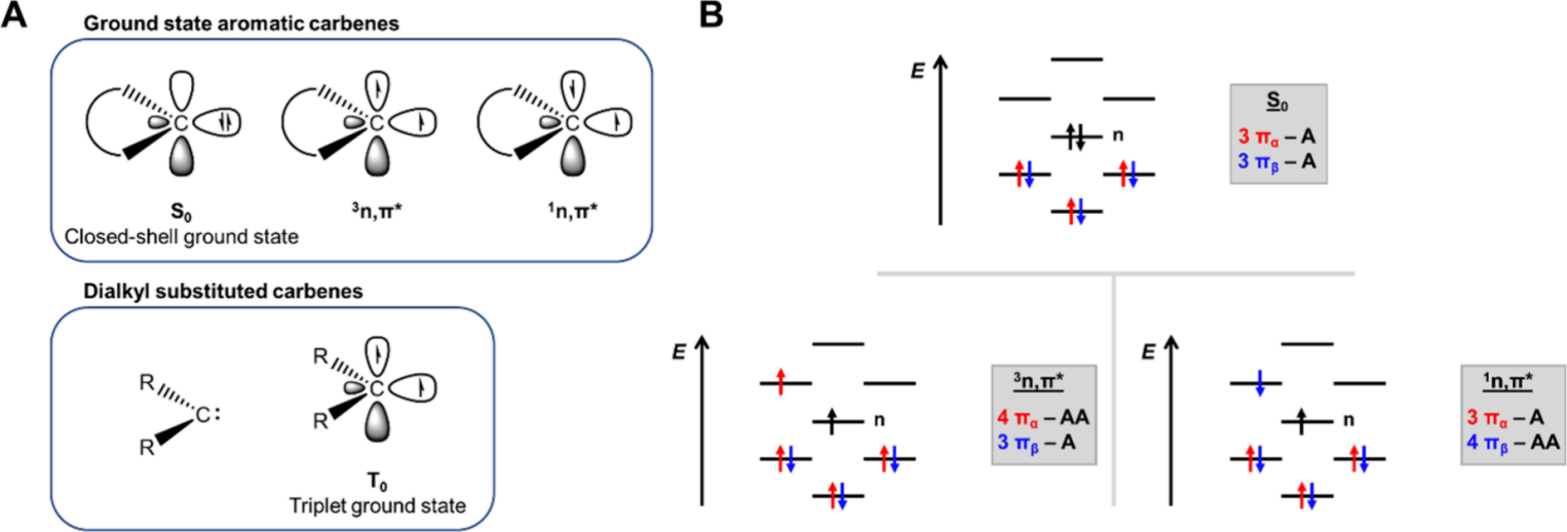
(A) Singlet and triplet states of ground state aromatic carbenes and the triplet ground state of dialkyl substituted carbenes, analogous to the ^3^ *n*,π* state of the former. (B) Illustration of the contributions of the π-electrons to the aromatic character of a 6π-electron species, as outlined by Mandado’s rule on (anti)­aromaticity for separate spins. The lone-pair orbital *n* is also included.

For a series of 6-MR heteroaromatics we earlier found a weak correlation between the energy gap between the ^3^ *n*,π* and ^3^π,π* states, Δ*E*(^3^π,π*–^3^ *n*,π*), and the extent of aromaticity in the ^3^ *n*,π* state.^13^ Herein, we explore (anti)­aromaticity in the lowest excited states of the molecules of Figure 1, and make use of Mandado’s rule which considers the contributions from α and β spins separately. This rule implies that 2*n* + 1 π-electrons of a specific spin (α or β) contribute to aromaticity, while 2*n* + 2 π-electrons of the other spin (β or α) contribute to antiaromaticity.^30^ For example, a heteroaromatic molecule with six π-electrons will in its *n*,π* state have three π-electrons of β spin and four π-electrons of α spin, as one electron was excited from an *n* to a π* orbital (Figure 2B). Indeed, the *n*,π* states, will have one π-electron more of one spin than of the other regardless of the spin multiplicity of the state (triplet or singlet). As a consequence, there will be a tug-of-war between one aromatic and one antiaromatic spin component, and an overall leaning toward aromatic or antiaromatic character will result if there is a nonzero residual between the two. This was also recently observed in a series of pro-aromatic radicals.^31^ Moreover, the benzene radical cation, with 2π_α_ and 3π_β_-electrons, have been reported to retain about one-third of the aromatic stabilization energy of benzene.^32^ In this context, the recent finding on an odd-electron annulene-within-annulene macrocycle^33^ is notable as it reveals that molecules can shift their (anti)­aromatic character through reorganization of their electronic structure so as to maximize aromaticity.

For 6-MR heteroaromatics, we observed that more electropositive heteroelements lead to higher aromatic character of the *n*,π* states.^13^ For molecules with more than one heteroatom, the extent of aromatic character of the residual was influenced by the relative positions of the two heteroatoms. However, we also found that the (anti)­aromatic character varies with aromaticity aspect considered (energetic, electronic, magnetic or geometric).

The S_0_ state aromaticity of *N*-heterocyclic carbenes (NHCs), such as **8**, was previously investigated by magnetic, electronic and energetic descriptors.^34–36^ These NHCs are of great importance in organic chemistry, even though the smallest are highly reactive and short-lived. In general, NHCs have slightly reduced aromatic character when compared to cyclopentadienyl anion (Cp^–^) as the cyclic π-conjugation of the carbenes involves dative interactions between an empty p_π_ atomic orbital (AO) at the carbene C atom and p_π_ lone-pair(s) at one (or two) heteroatoms. Yet, upon excitation to the *n*,π* state, the additional π-electron in the conjugated system will lead to a species with unclear (anti)­aromatic character.

Several questions need to be answered in order to decipher what factors influence the energetic order between the *n*,π* and π,π* state, i.e., Δ*E*(*n*,π*−π,π*). How does the aromatic or antiaromatic character of the residual in the *n*,π* state vary with ring size and heteroatoms adjacent to the carbene C atom? How does it vary if the sp^2^ hybridized carbene C atom is exchanged to a similarly hybridized (valence) isoelectronic heteroatom E = B^–^, N^+^, P^+^, or Si? These atoms have different electronegativities and abilities for π-orbital overlap with the neighboring p_π_ AO. Moreover, if the cause for the high excitation energy of *n*,π* states in 5-MR heteroaromatics is the more acute valence angles at the E atoms of these species, compared to those of 6-MRs, then what is the case in cyclopropenylidene, a 3-MR carbene which is 2π-electron Hückel-aromatic in S_0_?

The carbenes explored here are found in numerous areas. Cyclopropenylidene is a small and highly reactive compound of astrochemical relevance,^37–39^ e.g., detected in the atmosphere of Saturn’s moon Titan.^39^ Furthermore, some of the NHC species explored are mesoionic (so-called abnormal NHCs, aNHCs), i.e., carbenes for which one can only draw zwitterionic resonance structures (**3**, **10**, **12**, and **14**, Figure 1).^40–43^ These carbenes have recently found interesting usages in organic synthesis and catalysis.^41^ Thus, these carbenes themselves are important, but they also lead us to general conclusions on the character of the lowest few excited states of heteroaromatics.

## Results and Discussion

Our first focus is on the (anti)­aromatic character of the vertically excited *n*,π* states, as this provides for a more direct comparison with the aromaticity in S_0_ than the geometrically relaxed *n*,π* states. We assume that the α-component of the *n*,π* state is the (formally) antiaromatic (2*n* + 2)­π-electron component, while the β-component is the (formally) aromatic (2*n* + 1)­π-electron component of the *n*,π* state. Of these, the α-component will strive to relieve its antiaromatic character while the β-component will seek to retain its aromaticity, leading to a tug-of-war.

We primarily study the triplet *n*,π* state (^3^ *n*,π*) as it can be explored by a larger set of aromaticity indices at UDFT level than what is possible for the singlet *n*,π* state (^1^ *n*,π*). The singlets must be computed with either TD-DFT, which cannot be spin-separated, or costlier electron correlated wave function methods. We showed earlier that the ^1^ *n*,π* and ^3^ *n*,π* states of regular heteroaromatics mostly exhibit the same trends in their (anti)­aromatic character.^13^ For the (anti)­aromaticity assessments we apply one electronic aromaticity descriptor (the multicenter index, MCI)^44,45^ and one magnetic descriptor (magnetically induced current densities, MICD).^46–49^ MCI is used for all compounds, while MICD is applied to a selection. Both descriptors can be separated into α- and β-components. Additionally, one energetic descriptor (the isomerization stabilization energy, ISE)^50^ and one geometric descriptor (harmonic oscillator model of aromaticity, HOMA)^51^ were used for a few species in their geometrically relaxed ^3^ *n*,π* structures, although these descriptors cannot be spin-separated. For further details see the Computational Methods section.

The boron-containing compounds **5**, **15**, and **20** have *n*,Rydberg states as their lowest excited state according to gas phase computations. Yet, in a solvent, modeled implicitly via a polarizable continuum model, this state moves up in energy for **5** and **20** (Table S16). For **15**, the T_1_ state remains of *n*,Rydberg character but with the T_2_ state having *n*,π* character only 0.09 eV above. For the 3-MR compounds there are also several σ,π* and/or *n*,σ* states, in addition to the *n*,π* states, below the first π,π* state according to TD-DFT (Table S14). Here it is noteworthy that changes in relative energies of excited states due to solvent effects are not unexpected,^52–57^ in particular, in excited states that involve some sort of charge separation.^58,59^ However, the analysis of solvent effects on the relative energies of the *n*,π* and π,π* states is outside the scope of this work.

### 6-MR Carbenes, Analogues, and Isomers

Here, we first consider carbenes **2**–**4** which are pyridine (**1**) isomers, and then analyze **5** and **6** which are isoelectronic and isostructural with carbene **4**. In that part, we also explore **7** and **8**, heavier congeners of **4** and **6**. In line with earlier findings,^13^ we find that for **2**–**4** and **6**, the T_1_ and S_1_ states are both of *n*,π* character and of the same symmetry (Table 1). For **5**, T_1_ and S_1_ are both of *n*,Rydberg character, but with ^3^ *n*,π* merely 0.22 eV higher up. However, the energy gaps between the lowest ^3^ *n*,π* and ^3^π,π* states (Δ*E*(^3^π,π*–^3^ *n*,π*)) vary extensively, with the smallest found for **6** and the largest for **5**. This reveals that the electronegativity of the divalent E atom substantially impacts the order between these states, as also found for regular heteroaromatics.^13^ Nevertheless, particular caution should be taken when the calculated Δ*E*(π,π*–*n*,π*) is below approximately 0.3 eV and the energetic ordering should be considered ambiguous due to method-inherent uncertainty.^60^ However, such uncertainty does not significantly affect the observed trends. But what other factors play roles in this trend?

**Table 1:**
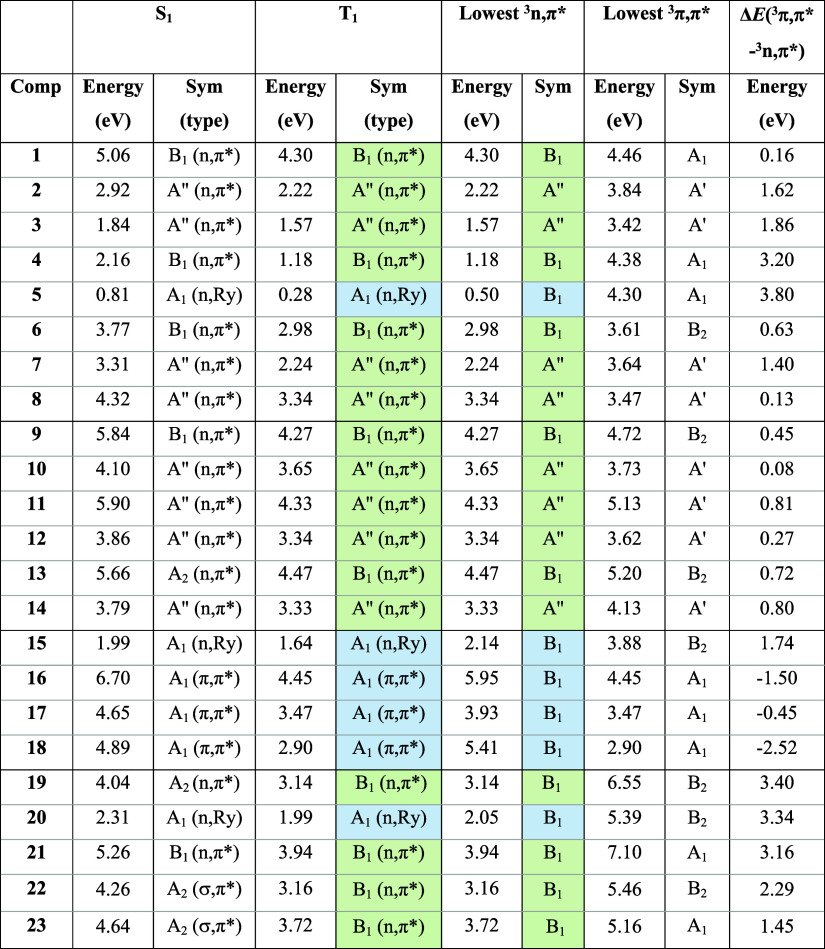
Vertical Excitation Energies of the S_1_, T_1_, Lowest ^3^ *n*,π*, and Lowest ^3^π,π* States, and Their Respective Symmetries^a^

a See Figure S3 in the SI for the orbitals involved and their respective symmetries. The rightmost column shows the energy difference between the lowest ^3^ *n*,π* and ^3^π,π* states. Energies calculated at (U)­CAM-B3LYP/6-311+G­(d,p) level in the gas phase. T_1_ states which are not of ^3^ *n*,π* character are highlighted in blue.

The MCI threshold for aromaticity in 6-MRs is set at half the MCI value of benzene in S_0_ (i.e., 0.0716/2 = 0.0358),^13^ and this value is used for both the S_0_ and ^3^ *n*,π* states of **1**–**6**. In S_0_, the three carbenes **2**–**4** each have low MCI values (Figure 3A), and only **2** should be described as aromatic according to our threshold. Indeed, the π-conjugated 6-MRs **2**–**4** with dative N–C π-bonds are isoelectronic and equivalent to azaborines, known to have reduced aromatic character due to the dative B–N π-bonds.^61^ However, the situation is different for the magnetically induced π-electron bond currents of **4** (9.4 nA/T, Figure 3B), constituting the main part of the total currents 9.3 nA/T (Table S7), since they approach those of benzene in S_0_ (11.5 nA/T).

**Figure 3:**
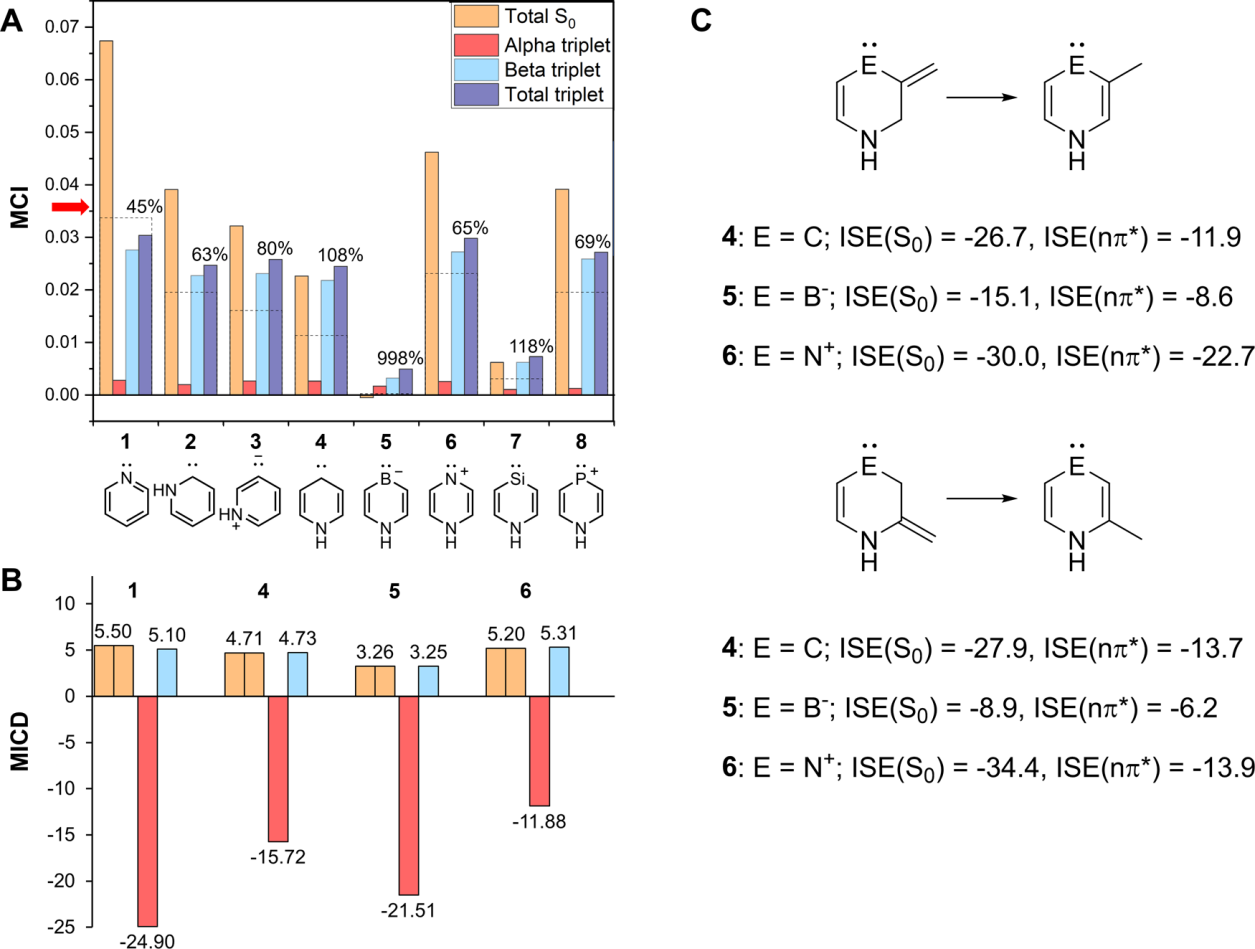
(A) MCI data (in a.u.) in the S_0_ and lowest vertical ^3^ *n*,π* states of the 6-MR compounds of Figure 1 (Table S1). The percentages correspond the total ^3^ *n*,π* value relative to that of S_0_, and the dashed lines mark 50%. The red arrow by the *y*-axis indicates the threshold values for aromaticity in 6-MRs (0.0358, see main text). (B) Average π-electron bond current strengths (in nA T^–1^) of **1** and **4**–**6** in their S_0_ (yellow bars) and ^3^ *n*,π* states (red bars show the α-contributions, and blue correspond to β). (C) Isomerization stabilization energies (ISE, in kcal/mol) of **4**–**6** in their S_0_ and vertical ^3^ *n*,π* states.

With regard to the energy aspect of aromaticity of **4** in S_0_, evaluated as the isomerization stabilization energy (ISE), at first glance, this seems similar to the MICD finding (Figure 3C) as ISE suggests clear aromatic character in S_0_, with values of −27.9 and −26.7 kcal/mol for the two isomeric methyl-substituted derivatives of **4**. These values are slightly lower than those of benzene and pyridine in S_0_ (−33.2 kcal/mol for toluene, and −29.4 to −33.5 kcal/mol for 2-, 3-, and 4-methylpyridine with B3LYP/6-311+G­(d,p)).^54^ However, a closer look at the geometries and electron delocalizations of the compounds show that these values are exaggerated (see Section 2.4 of the SI).

In their lowest ^3^ *n*,π* states, the MCI_β_-components of **2**–**4** are raised significantly when compared to S_0_, revealing increased aromatic character of the 3π_β_-electron component. Furthermore, the MCI­(^3^ *n*,π*)_tot_ values of the three carbenes are similar, which for **4** means that the MCI_β_-component of ^3^ *n*,π* alone is nearly as large as the MCI­(S_0_)_tot_ (Figure 3A). This indicates a nearly doubled aromatic character of the π_β_-component of the ^3^ *n*,π* state of **4** when evaluated based on the electronic aromaticity aspect. On the other hand, the MCI_α_-components in the ^3^ *n*,π* states of **1** and **2–** **4** are small and similar, revealing antiaromatic 4π_α_-electron character. Still, and as just mentioned, the combined MCI_α_ and MCI_β_ components of **4** in its ^3^ *n*,π* state is larger than the MCI of the S_0_ state, and thus, this carbene has an ^3^ *n*,π* state which leans more toward aromatic character than the S_0_ state. Despite this, it is important to stress that **4** in ^3^ *n*,π*, according to MCI, should be labeled as nonaromatic as its MCI­(^3^ *n*,π*)_tot_ value is below our aromaticity threshold of 0.0358 (Figure 3A).

The spin-separated MICD results for the ^3^ *n*,π* state of **4** reveal that the magnetically induced bond currents of the 3π_β_-electrons remain essentially the same as in S_0_ (Figure 3B), while those of the 4π_α_-manifold are markedly paratropic (antiaromatic). However, we earlier found that magnetic descriptors for triplet *n*,π* states of heteroaromatics often overestimate the antiaromatic contributions when compared to electronic descriptors.^13^ Thus, to get insights of the cause of the values computed with magnetic aromaticity descriptors for species with odd total numbers of π-electrons one must analyze the α- and β-spin components separately. Considering the energy aspect of (anti)­aromaticity, the two ISE values of **4** in the ^3^ *n*,π* state are negative (Figure 3C), indicating that the aromaticity of the 3π_β_-electron component is stronger than the antiaromaticity of the 4π_α_-electron component, unless the latter component is nonaromatic contributing with near-zero ISE value.

Although there are several factors that influence excitation energies, for pyridine and carbenes **2**–**4** one may argue that the change in (anti)­aromatic character when going from the S_0_ to the ^3^ *n*,π* state is related to the vertical *E*(^3^ *n*,π*). Pyridine, with an *E*(^3^ *n*,π*) of 4.30 eV (Table 1), has an indisputably aromatic S_0_ state, providing for stabilization. In contrast, its ^3^ *n*,π* state tends toward antiaromatic character leading to destabilization. Carbene **4** which is nonaromatic in both S_0_ and ^3^ *n*,π* according to MCI, although tending more toward aromaticity in ^3^ *n*,π* than in S_0_, has an *E*(^3^ *n*,π*) of merely 1.18 eV. The relative energies of these two species in S_0_ and in ^3^ *n*,π* are in support of these relative (anti)­aromaticity characters because **4** is less stable than **1** by 2.60 eV in S_0_ but more stable than **1** in ^3^ *n*,π* by 0.51 eV. For **2** and **3**, the difference in aromaticity between the S_0_ and ^3^ *n*,π* states given by differences in MCI values, ΔMCI­(S_0_–^3^ *n*,π*), as well as their *E*(^3^ *n*,π*) are both in between the corresponding values of **1** and **4**.

The (anti)­aromatic character of isomers **2**–**4** are to some degree reflected in their relative energies in the two states. The aromatic character in S_0_ decreases gradually when going from **2** over **3** to **4**, and it is reflected in the relative S_0_ energies, with **2** being lower in energy than the other two by 0.69–0.73 eV. Going to the ^3^ *n*,π* state, the MCI­(^3^ *n*,π*)_tot_ values for all three species are rather similar, with that of carbene **3** (mesoionic in S_0_) being modestly higher compared to the other two carbenes. The energies also vary less in this state and combined with the S_0_ state energies this leads to the largest *E*(^3^ *n*,π*) for **2** (2.22 eV) and the smallest for **4** (1.18 eV). Another noteworthy aspect is the geometric relaxation in the ^3^ *n*,π* states. Carbene **4** with an MCI­(^3^ *n*,π*) residual that tends toward an aromatic character is essentially planar, except for a minute pyramidalization at the N atom (H–C–N–H dihedral angle of ∼10°). The HOMA value of **4** is 0.85 in S_0_ and 0.80 in its relaxed ^3^ *n*,π* state (Table S8).

We next relate carbene **4** to the isoelectronic species **5** and **6** with the divalent C atom replaced by, respectively, formally divalent B^–^ and N^+^ atoms with lone-pair electrons at these atoms. Thereby, it becomes apparent to what extent the aromatic character can vary with electronegativity, both in the S_0_ and in the ^3^ *n*,π* state (see below). It also becomes apparent how the electronegativity impacts on the order between the *n*,π* and π,π* states because **6,** with the electronegative N, has the smallest Δ*E*(^3^π,π*–^3^ *n*,π*). Also, the excitation energies of **4**–**6** vary extensively, as **6** has an *E*(^3^ *n*,π*) of 2.98 eV while for **5** it is merely 0.50 eV. Noteworthy, the UCCSD value of *E*(^3^ *n*,π*) for **5** is 0.56 eV, in good agreement with the UDFT value. Thus, the *E*(^3^ *n*,π*) of the three carbenes **2**–**4** are intermediate between those of **5** and **6**. Compound **6** also illustrates the relationship between formally aromatic carbenes and regular heteroaromatics as this compound is more appropriately described as protonated pyrazine than by a resonance structure with a divalent N^+^ atom (Figure 1). This explains why **6** has a high aromatic character in S_0_ according to MCI, MICD, and ISE (Figure 3).

Compound **5** has an exceptionally low MCI­(S_0_) value as this molecule will avoid aromaticity since an aromatic resonance structure places an additional negative charge on the formally negatively charged B atom (see Figure S5). The MICD results for **4**–**6** in S_0_ (Figure 3B) are in line with the MCI results, the difference is, however, much smaller. When compared to the bond currents of benzene in S_0_ (5.77 nA/T per spin), **4**, **5**, and **6** with bond currents at 3.26 nA/T per spin, or higher, should all be regarded as aromatic.

For the ^3^ *n*,π* states of **4**–**6**, we first consider **6**. According to MCI, this compound has a residual in its ^3^ *n*,π* state which tends toward aromaticity at about 65% of the S_0_ state (Figure 3A), and with the MCI_β_ component larger than half the MCI­(S_0_) value. The MICDs reveal slightly stronger diatropic bond current of the π_β_-electrons than in S_0_ while the paratropic π_α_-electron current is the smallest among **4**–**6**. Finally, the ISE values that correspond to the reactions leading to the 2- and 3-methyl substituted **6** also indicate aromatic character (Figure 3C). The ISE of **6** in the ^3^ *n*,π* state, with three π_β_-electrons, is about half that of S_0_ (if, among the two, we consider the reaction with the lowest ISE). Similar as for **4** in its ^3^ *n*,π* state, this should indicate that the aromatic character of the 3π_β_-component of **6** is stronger than the antiaromatic character of the 4π_α_-component. Furthermore, when the geometry of **6** is relaxed in its ^3^ *n*,π* state the molecule stays planar, a feature that is in line with aromaticity.

With regard to **5** in its ^3^ *n*,π* state, the negative charge at the B atom is reduced from −0.21 to 0.01 *e* (NBO charges) since one α-electron is moved from the sp^2^ orbital (lone-pair) of boron and placed in the delocalized π* orbital. This leads to a massive increase in the MCI value by ∼1000%, yet, it goes from being nearly zero to a value which is still very low, indicating lack of aromaticity. This is in line with the MICDs for **5** as it exhibits the strongest paratropic (antiaromatic) 4π_α_-electron contribution among **4**–**6**, while the 3π_β_-contribution remains as in S_0_. The paratropic 4*n*π_α_-contributions of **4** in its ^3^ *n*,π* state are intermediate between those of **5** and **6**. Finally, the ISE values for the ^3^ *n*,π* state of **5** are slightly negative (Figure 3C), suggesting that the 3π_β_-component has an increased aromatic character when compared to the S_0_ state but that the compound is clearly nonaromatic in this state.

By going to the heavier Si congener of **4**, i.e., **7**, one can note that the *E*(^3^ *n*,π*) is considerably higher than for **4** (Table 1), while the relationship is the opposite for *E*(^3^π,π*) of the two compounds. For *E*(^3^ *n*,π*), the finding should stem from a less strong two-electron Coulomb repulsion in the larger *n*(Si) orbital than in the *n*(C) orbital. This resembles what has earlier been reported for SiH_2_ vs CH_2_,^62^ and it leads to a higher relative *E*(^3^ *n*,π*) when compared to *E*(^3^π,π*) in **7** than in **4**. One can note a similar relationship in the *E*(^3^ *n*,π) of **8** when compared to that of **6**. Interestingly, **7** exhibits a very low degree of aromaticity according to MCI, both in S_0_ and in ^3^ *n*,π* (Figure 3A). Compound **8** in S_0_, on the other hand, should be labeled as aromatic, although it is below the threshold for this label in ^3^ *n*,π* despite that the residual leans toward aromaticity.

### 5-MR Carbenes and Analogues

We next explored 5-MR carbenes and heteroelement analogues, with the apparent difference to the 6-MR species being the smaller bond angles at the divalent C and E atoms. The 5-MR carbenes include both normal and mesoionic (abnormal) NHCs **9**–**14**, which, in line with our hypothesis, have T_1_ and S_1_ states with *n*,π* character (Table 1), in contrast to regular 5-MR heteroaromatics which have ^3^π,π* states as these (see Introduction). From Table 1, one sees that the Δ*E*(^3^π,π*–^3^ *n*,π*) gap is small only for the mesoionic **10** and **12** (0.08 and 0.27 eV, respectively). Importantly, the state symmetries are the same for T_1_ and S_1_ (Table 1), which shows that our analysis is valid also for the directly accessible singlet excited states.

We further explored the heteroelement compounds **15**–**18** which are valence isoelectronic and isostructural with **9** but with formally divalent E atoms with electronegativities that differ from that of C, and which π-conjugate via either 3p_*z*_ AOs (Si and P) or 2p_*z*_ AOs (B and N). In contrast to the T_1_ states of **9**–**14**, those of **16**–**18** are of π,π* character, whereas **15** with E = B has a T_1_ state of *n*,Rydberg character. In **15**, the T_2_ and S_2_ states are instead of *n*,π* character while the lowest π,π* states are higher in energy. Similar to **9**–**14**, the S_1_ states of **15**–**18** are of the same type and symmetry as the T_1_ states.

By comparing the differences in the Δ*E*(^3^π,π*–^3^ *n*,π*) gap between analogous 6-MR and 5-MR species (Table 1), we get further support for the hypothesis posted in the Introduction. For carbenes **2**–**4** and **9**, the gaps are found at 1.62–3.20 and 0.45 eV, respectively, for the two heteroelement analogues with E = N (**6** and **16**) they are 0.63 and −1.50 eV, and finally for the two analogues with E = B (**5** and **15**) they are 3.80 and 1.74 eV. Thus, the gap shrinks for all three pairs when the ∠N–E–N angle decreases, and with E = N (**16**) it even becomes negative, i.e., this compound has a T_1_ state of π,π* character. The smaller bond angles in the 5-MR than in the 6-MR compounds (e.g., 103.8° in **16** and 118.8° in **6**) are reflected in lower ε_n_, and consequently, higher *E*(^3^ *n*,π*) in the first class of compounds (Table 1, e.g., 5.94 eV in **16** and 2.98 eV in **6**). Indeed, when altering the ∠N–C–N angle in H_2_N–C–NH_2_ from 108° to 120°, representing the angles for perfect pentagons and hexagons, the ^3^ *n*,π* and ^1^ *n*,π* states move down in energy by 0.37 and 0.52 eV while the ^3^π,π* and ^1^π,π* states are increased by 0.34 and 0.43 eV (Figure 4). Yet, there are also other factors that can impact such as the electronegativity of the atoms adjacent to the E atom.

**Figure 4:**
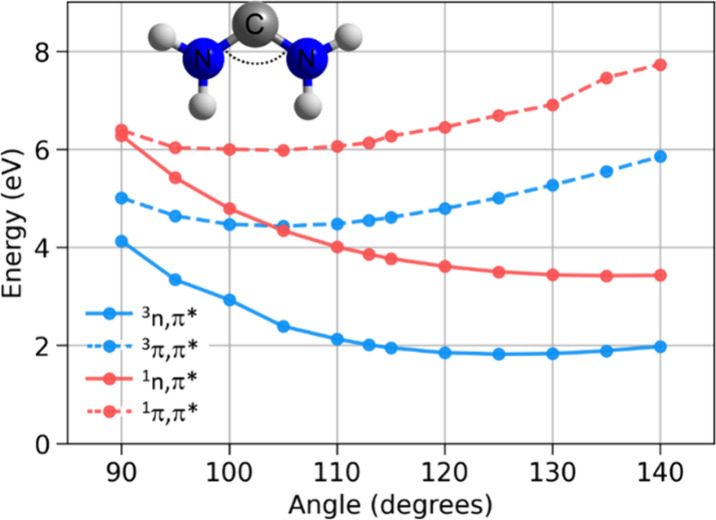
Variation in vertical excitation energies as a function of ∠N–C–N angle in diaminocarbene.

Now, going from **9** to the *N*-heterocyclic silylene **17**, the T_1_ state changes character from ^3^ *n*,π* to ^3^π,π*, a change that stems from two factors. The energies of the *n* orbitals (ε_*n*_) centered on the divalent Si vs C provide an explanation as the ε_*n*_(Si) of **17** is lower than the ε_*n*_(C) of **9** (−8.21 vs −7.72 eV). This effect should stem from a less strong two-electron Coulomb repulsion in the larger *n*(Si) orbital than in the *n*(C) orbital,^66^ similar as discussed for the 6-MR **7** vs **4**. This leads to a higher relative *E*(^3^ *n*,π*) when compared to *E*(^3^π,π*) in **17** than in **9** as is clear from the Δ*E*(^3^π,π*–^3^ *n*,π*) values of −0.45 and 0.45 eV, respectively (Table 1). Indeed, one sees the same trend in the Δ*E*(^3^π,π*–^3^ *n*,π*) of **16** and **18** with formally divalent N^+^ and P^+^. In addition, there is also the effect of the ∠N–E–N angle as becomes apparent when comparing **7** with **17**, and **8** with **18**.

With regard to the aromatic character, the MCI threshold value for the aromaticity of 5-MRs is set at 0.0338, taken as half the MCI value of the cyclopentadienyl anion (Cp^–^) in its S_0_ state.^13^ When compared to this, each of **9**–**14** in their S_0_ states should be categorized as aromatic or weakly aromatic (Figure 5A), while this is only the case for **16** and **18** among the heteroatom analogues (Figure 5B). Among the carbenes in S_0_, the aromaticity increases successively with the number of N atoms, and for **13** and **14** the MCI­(S_0_) values approach that of Cp^–^. The MICDs of **9** in S_0_ reveal a diatropic π-bond current of 7.5 nA/T, which is weaker than that of Cp^–^ (11.1 nA/T). This difference is larger than earlier found with NICS, as NICS(1)_*zz*_ values of −28.58 and −33.56 were reported for **9** and Cp^–^, respectively.^34^ Ring current contributions from the σ-electrons can be a reason for this (see Table S7). The weak aromatic character of **9** in S_0_ is supported by an ISE value of −14.7 kcal/mol. The MICDs reveal diatropic ring currents for **15** and **16** (heteroelement analogues of **9**) with total π-bond currents of 5.8 and 9.0 nA/T, respectively (Figure 5C, for **16**). However, their ISE values in S_0_ span from nonaromatic (**15**) to strongly aromatic (**16**) (Figure 5D).

**Figure 5:**
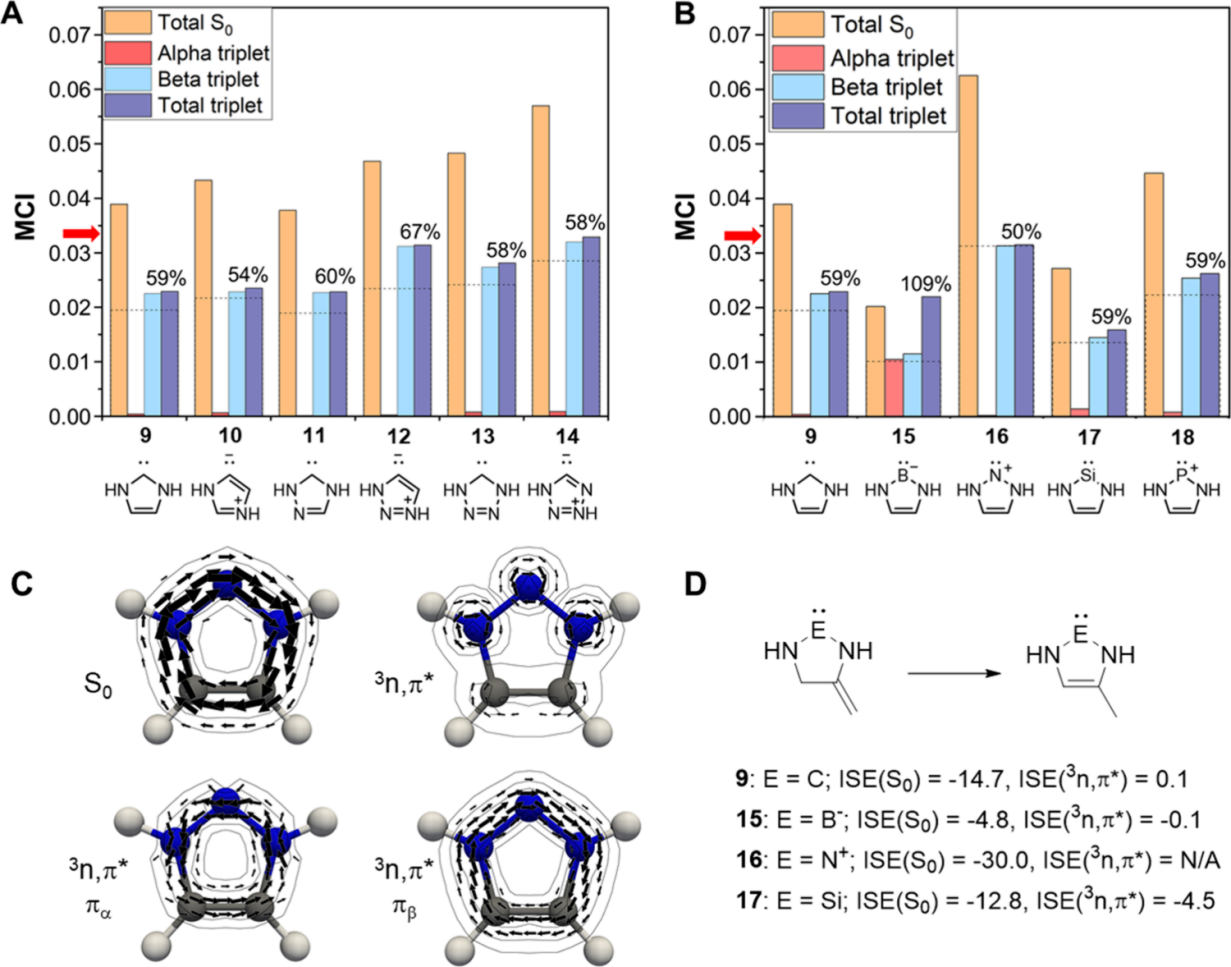
MCI data (in a.u.) of (A) the 5-MR carbenes in the third row of Figure 1, and (B) carbene **9** and its heteroelement analogues shown on the forth row of Figure 1 (Table S1). The percentages correspond to the total ^3^ *n*,π* value relative to that of S_0_, and the dashed lines mark 50%. The red arrow on the vertical axis in each plot represents the threshold MCI value for aromaticity of 5-MRs (0.0338). (C) MICD plots of **16** in S_0_ and ^3^ *n*,π*, respectively, with the π_α_- and π_β_-ring currents of the ^3^ *n*,π* state plotted. (D) ISE values of **9**, **15**–**17** in their S_0_ and ^3^ *n*,π* states. The ISE of **16** in ^3^ *n*,π* cannot be determined due to lack of symmetry of the nonaromatic isomer.

Upon excitation of an electron from the *n* orbital to a π* orbital in the 5-MR species, the relief of intramolecular Coulomb repulsion should be smaller than in the 6-MR species because now the seven π-electrons of the *n*,π* state are distributed over only five p_π_ AOs instead of six. In line with this, the excitation energies to the ^3^ *n*,π* states of the three 6-MR carbenes **2**–**4** are lower than those of the 5-MR carbenes **9**–**14** (Table 1). Moreover, the MCI values of **2**–**4** in their ^3^ *n*,π* states are 67–109% of the S_0_ values, while for the 5-MR carbenes **9**–**14** they are 54–67%.

Four of the six carbenes **9**–**14** in their ^3^ *n*,π* states, should be labeled as nonaromatic based on the electronic MCI index (Figure 5A). Only **12** and **14** have total MCI values close to our threshold for aromaticity. Yet, similar to the S_0_ state, there is some increased aromatic character when going from **9** to **14**, and the mesoionic carbenes (**10**, **12**, and **14**) have slightly higher aromatic character than the corresponding normal NHC isomers. The MICD computation for the ^3^ *n*,π* state of **9** reveal an overall paratropic ring current with a dominating paratropic π_α_ bond current of −9.2 nA/T and a π_β_ diatropic one of 3.8 nA/T, and the situation is the same for **13** and **14** but with slightly stronger paratropic α-currents. With regard to the energy aspect of aromaticity, the ISE value of **9** in ^3^ *n*,π* (0.1 kcal/mol) signals a perfectly nonaromatic character, a feature that is possible either (i) if the antiaromatic 4π_α_-electron component is equally destabilizing as the aromatic 3π_β_-component is stabilizing, or (ii) if both components are nonaromatic.

Among **15**–**18**, i.e., the heteroelement analogues of **9**, we first regard **16**. In Figure 5B one sees that its aromatic character according to MCI is reduced extensively when going from S_0_ to ^3^ *n*,π*. The ^3^ *n*,π* state of **16** is clearly nonaromatic as the aromatic MCI_β_ component is identical to that of the S_0_ state while the antiaromatic MCI_α_ component has a very low value signaling strong antiaromatic character. The marked reduction in aromaticity is supported by the MICD result because the individual π_α_- and π_β_-electron ring currents are, respectively, paratropic and diatropic to about equal extents (Figure 5C), leading to annihilation of a ring current as seen in the plot of the total current density.

For **15**, the situation is drastically different, as the MCI_α_ value indicates extensive relief of 4π_α_-electron antiaromaticity. We attribute the reduced α-antiaromaticity in **15** to the fact that the promoted α-electron goes to a diffuse π-orbital that could be classified as a Rydberg orbital (Figure S4), and, consequently, the effective π_α_-electrons are closer to three than four, resulting in almost equal MCI_α_ and MCI_β_ values. Another interesting finding in this context is that the B-containing **15**, viewed relative to carbene **9**, exhibits a markedly stronger aromatic character in both its S_0_ and ^3^ *n*,π* states than the B-containing **5** viewed relative to 6-MR carbene **4**. Still, **15** in its ^3^ *n*,π* state is nonaromatic according to ISE (Figure 5D), and this is supported by MICD as its π-bond current is modestly diatropic (4.0 nA/T, with the π_α_- and π_β_-components at 1.0 and 3.0 nA/T).

Now, is there a relationship between the difference in aromatic character between the S_0_ and ^3^ *n*,π* states, as assessed by ΔMCI­(S_0_–^3^ *n*,π*), and the *E*(^3^ *n*,π*)? For this analysis we excluded the mesoionic **10**, **12**, and **14** since they are extensively destabilized in S_0_ due to their zwitterionic character (they are less stable in S_0_ than their isomeric normal NHCs **9**, **11**, and **13** by 0.90–1.45 eV). Additionally, we included F and SiH_3_ substituted versions of five of the remaining seven compounds (**9** and **15**–**18**). Indeed, a plot of *E*(^3^ *n*,π*) vs ΔMCI­(S_0_–^3^ *n*,π*) for the 17 compounds gives an *R* ^2^ = 0.75 for a linear regression (Figure 6A), suggesting a connection between the difference in extent of (anti)­aromaticity in the two states and the vertical energy difference between them. This is the case despite the fact that the range in *E*(^3^ *n*,π*) is nearly 5 eV, and that the substituent effects inevitably introduced with the additional species likely affect the excitation energies also via σ-withdrawing or -donating abilities. In fact, when the compounds are grouped by their substitution, the correlation is further improved (*R* ^2^ values between 0.88–0.92, Figure S6). On the other hand, there is no correlation for the Δ*E*(^3^π,π*–^3^ *n*,π*) gap plotted against the MCI­(^3^ *n*,π*) values (Figure 6B). This reveals that the energy gap between the ^3^ *n*,π* and ^3^π,π* states also depends on the extent of Baird-antiaromatic character in the π,π* state leading to a relative destabilization and increase of the gap.

**Figure 6:**
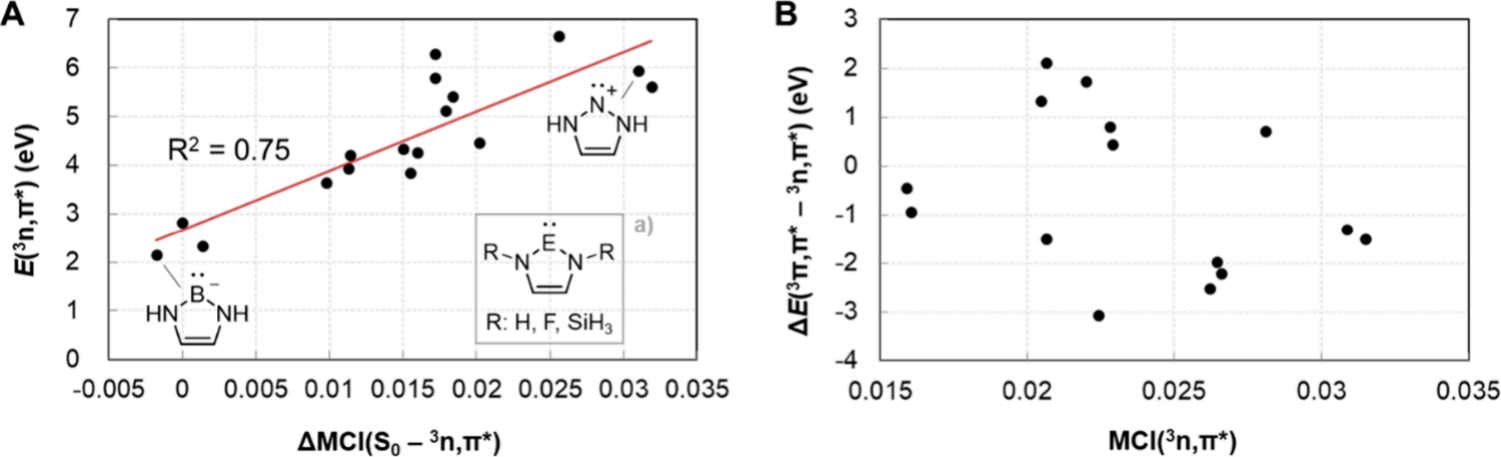
Correlations between (A) the vertical *E*(^3^ *n*,π*) and the S_0_–^3^ *n*,π* (anti)­aromaticity difference as assessed by ΔMCI­(S_0_–^3^ *n*,π*), and (B) the energy difference between the lowest ^3^π,π* and ^3^ *n*,π* states (Δ*E*(^3^π,π*–^3^ *n*,π*)) and the (anti)­aromaticity of ^3^ *n*,π* assessed by MCI­(^3^ *n*,π*). ^a^Additionally, compounds **11** and **13** with, respectively, three and four N atoms in the ring are included in the plot.

### 3-MR Carbenes and Analogues

While the 6-MR and 5-MR species are all 6π-electron compounds in S_0_, the 3-MR species have two π-electrons, allowing us to test the generality to species with different number of π-electrons. Of 3-MR species we explored cyclopropenylidene (**19**), the isoelectronic B^–^ and N^+^ analogues **20** and **21**, and the heavier Si and P^+^ analogues **22** and **23**. The analysis of these species is, however, primarily given in the Supporting Information.

In brief, it is known that the two *n*,π* states of **19** (the first B_1_ and A_2_ states) are lowest in energy among both the singlet and triplet excited states,^63^ and also **20** and **21** have lowest valence excited triplet states of ^3^ *n*,π* character with B_1_ symmetry. Our computed *E*(^3^ *n*,π*) of **19**–**21** range from 2.05 eV for **20** to 3.94 eV for **21** (Table 1). Here, the fact that the 3-MR species, with even more acute angles at the E atoms than the 5-MR species, have *n*,π* states as their lowest excited states is interesting as it is contradictory to our hypothesis. Clearly, additional factors impact the relative order of the *n*,π* and π,π* states than the valence angle at E.

The lowest ^3^π,π* states of **19**–**21** are higher in energy than the ^3^ *n*,π* states by more than 3 eV, and for **22** and **23** by 1.4–2.3 eV. As they are found at energies 5.16–7.10 eV (Table 1), higher than the lowest ^3^π,π* state of ethylene (4.47 eV), it suggests strongly destabilizing Baird-antiaromatic character. For **19**, the latter is illustrated by the fact that there are more than five states of ^3^n,π*, ^3^σ,π*, and/or ^3^ *n*,σ* character below the first π,π* state according to TD-DFT (Table S14). Obviously, the order between the *n*,π* and π,π* states also depends on the Baird-antiaromatic destabilization of π,π* states, a feature that is strongest for small rings, and attenuated when they expand.^64^

As the threshold for aromaticity of these species, we use half of the MCI value of the cyclopropenium cation in S_0_, i.e., 0.1968. In S_0_, **19** and **21** have a significantly stronger aromaticity than the other three 3-MRs (Figure 7A), approaching that of the cyclopropenium cation (0.3937). Indeed, **21** can be viewed as an azacyclopropenium cation (Figure 1). On the other hand, according to the computed MICD (Figure 7B) **20** exhibits nearly as strong aromaticity as **19** and **21**. For the analysis of 3-MRs based on MCI data, it should be stressed that σ-contributions to the (anti)­aromatic character were found to be significant (see Section 1 of the SI).

**Figure 7:**
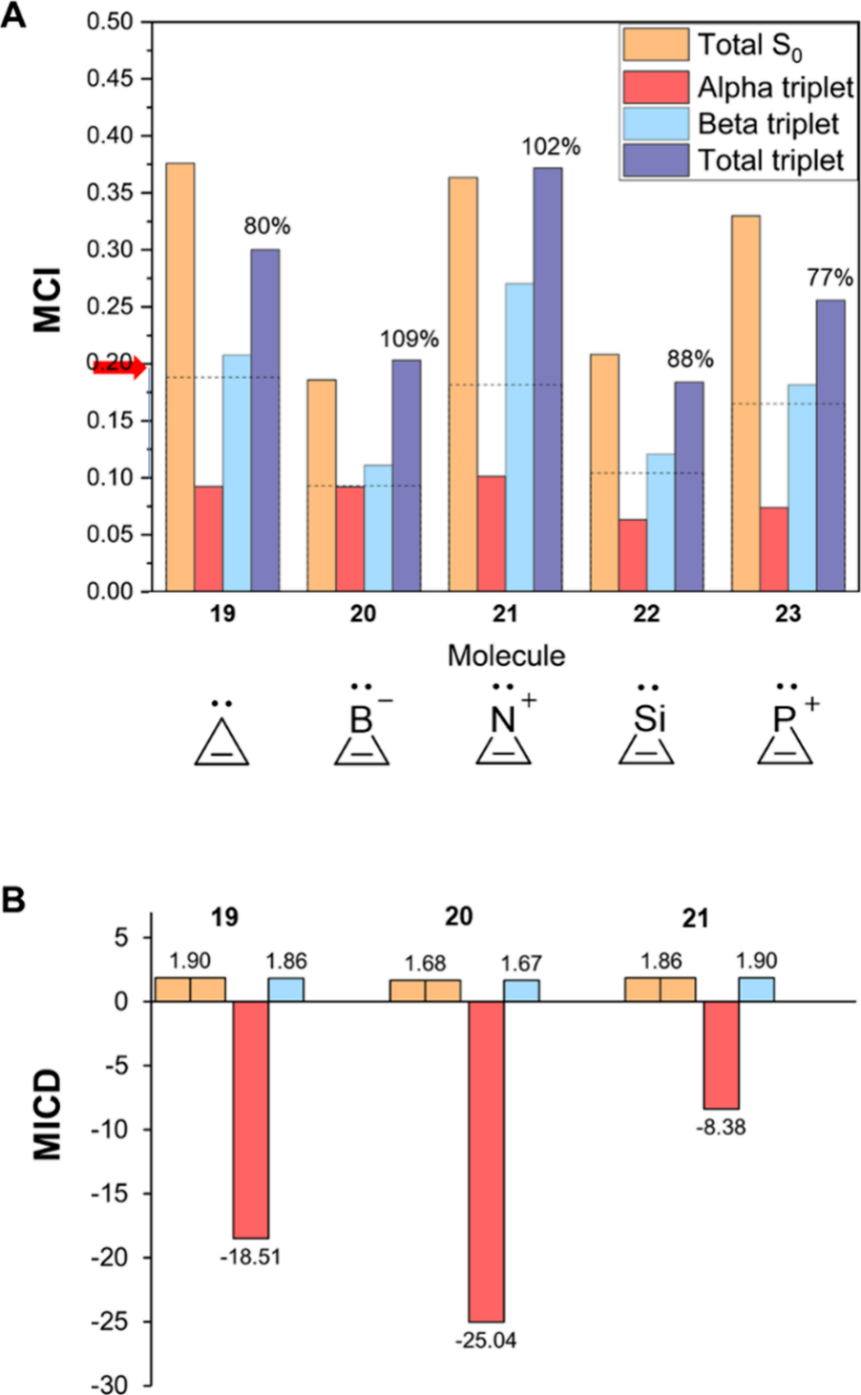
(A) MCI data (in a.u.) for the 3-MR compounds of Figure 1 (Table S1). The percentages correspond the total ^3^ *n*,π* value relative to that of S_0_, and the dashed lines mark 50%. The red arrow on the vertical axis represents the threshold MCI value for aromaticity of 3-MRs (0.1968). (B) Average π-electron bond current strengths (in nA T^–1^) of the 3-MRs of Figure 1 in their S_0_ (yellow bars) and ^3^ *n*,π* states (red bars show the α-contributions, and blue correspond to β).

In the lowest ^3^ *n*,π* state, **19** has a residual based on MCI with significant aromatic character (80%) when compared to the value of the S_0_ state (Figure 7A). Conversely, the MICD strengths for the ^3^ *n*,π* state of **19** (Table S7) show that π_β_-electron currents in the *n*,π* state stay the same as in the S_0_ state, but the π_α_-electron currents make a very strong paratropic contribution in line with relatively small HOMO–LUMO gap among the α-spin electrons (Table S7).

By changing the divalent C and N^+^ atoms of **19** and **21** to divalent Si or P^+^ centers (**22** and **23**), the p_π_-orbital overlap decreases, with the consequence that the S_0_ state aromaticity as well as the *E*(^3^π,π*) values decrease (Table 1). Still, the two lowest triplet states are of *n*,π* character (Table S11). Furthermore, the residual clearly leans toward aromaticity, with the total MCI values of the T_1_ state (the ^3^B_1_ state) at 77–88% of the values in the S_0_ state. The strong aromatic character of the lowest ^3^ *n*,π* states of all 3-MRs is also in line with the low *E*(^3^ *n*,π*), which are found at 3.14 (**19**), 2.05 (**20**), 3.94 (**21**), 3.16 (**22**), and 3.72 (**23**) eV, remarkably low for such small species (Table 1). However, it should be noted that there is no relationship between the ΔMCI­(S_0_–^3^ *n*,π*) and *E*(^3^ *n*,π*), as there was for the 5-MR species (Figure 6A). A tentative cause is the large σ-contributions to the MCI_α_ values.

In this context, the term “adaptive aromaticity” has recently been used to describe the aromaticity of molecules which according to computations show aromatic character in several electronic states, often the closed-shell singlet ground state and the lowest triplet state.^65,66^ Our analysis here, however, shows that aromaticity and antiaromaticity in states with unequal numbers of π_α_- and π_β_-electrons stem from residuals between these two components, that lean toward either aromaticity or antiaromaticity (see further in Section 1 of the SI). Thus, what has been labeled as “adaptive aromaticity” is not a new and unique form of aromaticity.

### Toward a Systematics for *n*,π* States of Heteroaromatics

One question that has driven this study is which heteroaromatics have *n*,π* states as their lowest excited states and which ones have π,π* states as these? We have seen that the electronegativity of the heteroatom as well as the valence angle at this atom, determined by ring-size, are important factors. But also other factors are influential such as the spatial extent of the *n* orbital, exemplified by silylenes vs carbenes, and the degree of (anti)­aromatic character of the residual in the *n*,π* state. Here, we summarize and further discuss the relationships between six different factors that impact on the order between the *n*,π* and π,π* states.

A first factor, not discussed explicitly above, is the number of π-orbitals set by the size of the π-conjugated cycle and the energy gap between the π-HOMO and π-LUMO. Obviously, the excitation energies of a 3-MR species with only three π-MOs should not be compared with those of a 7-MR species with seven π-MOs as the latter has a smaller energy gap between the π-HOMO and π-LUMO. The gap in the π-orbital energies of **19** and **24** (cycloheptatrienylidene, constrained to *C*_2v_ symmetry; Figure 8A) reflect the HOMO–LUMO gaps of *c*-C_3_H_3_^+^ and *c*-C_7_H_7_^+^ from Hückel MO theory (3.00β and 1.70β, respectively). The excitation energy of the lowest *n*,π* state relative to that of the first π,π* state depends on where the *n* orbital is found in energy relative to the π and π* orbitals. The *n* orbital of cyclic carbenes is found above the π-HOMO, but it will be different for other heteroaromatics depending on the electronegativity of E.

**Figure 8:**
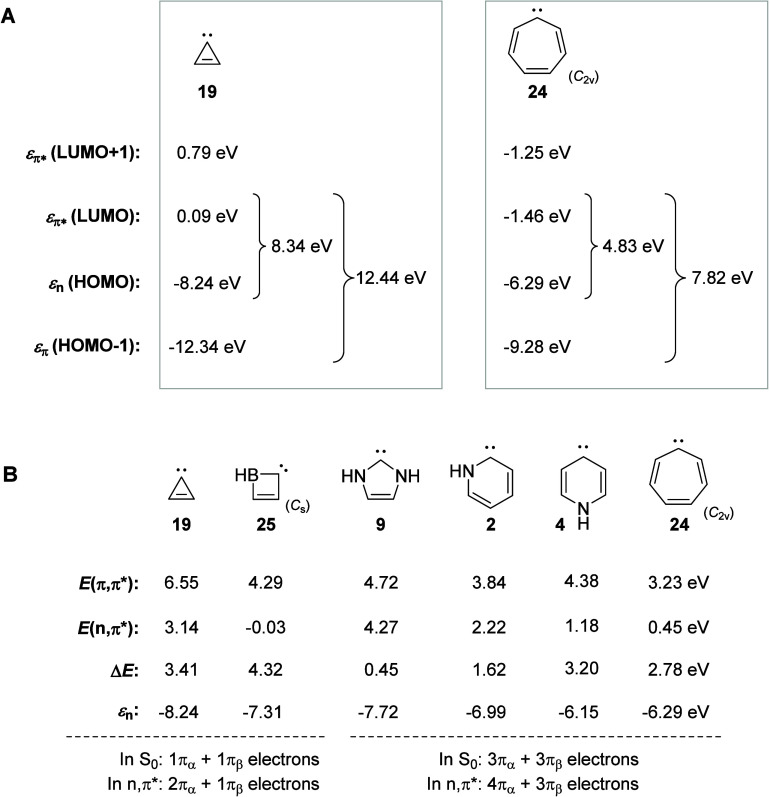
(A) Orbital energies and characters of the highest two occupied and the two lowest MOs of cyclopropenylidene and cycloheptatrienylidene, the latter constrained to *C*_2v_ symmetry. (B) Computed vertical excitation energies of the lowest triplet π,π* and *n*,π* states, the energy differences, and the energies of the *n* orbitals for selected compounds.

Accordingly, a second factor that impacts is the electronegativity of the heteroatom E (χ_E_). It is clear that the higher the electronegativity the lower in energy is the *n* orbital, and e.g., O-containing heteroaromatics of a given ring size will have higher *E*(*n*,π*) than the analogous N-containing ones (e.g., pyrylium ion vs pyridine). Yet, as revealed herein, and in our earlier study,^13^ ε_*n*_ does not only depend on the heteroelement E; it also depends on the valence angle at the E atom, which varies with the ring size. Hence, the valence angle is the third factor. As the *n* orbital is more localized to the E atom than the π and π* orbitals are, it will be more affected by the ∠X–E–X angle. As seen in Figure 8A, the ε_*n*_ moves up in energy by ∼2 eV when going from the 3-MR carbene **19** to the 7-MR carbene **24**. A comparison of the 6π-electron cycles **9**, **2**, **4**, and **24**, reveals that *E*(^3^ *n*,π*) decreases as the valence angle at E increases (Figure 8B), in line with findings for the acyclic H_2_N–C–NH_2_ carbene (Figure 4).

In the section on the 5-MR species, we analyzed the differences in the Δ*E*(^3^π,π*–^3^ *n*,π*) gaps for 5-MR vs 6-MR 6π-electron species with B, C, and N as (formally) divalent atoms, and we found that the gaps are larger for the 6-MR species than for the 5-MR ones. The same trend is seen for 2π-electron species as the gap between the ^3^ *n*,π* and ^3^π,π* states decreases when going from the 4-MR **25** (constrained to *C*_s_ symmetry) to the 3-MR **19** (Figure 8B). In general, the energy gap between the *n*,π* and π,π* states gets smaller, or even inverts to negative, with a more acute angle at the heteroatom E. However, it is noteworthy that one cannot compare species with different numbers of π-electrons.

The fourth factor that influences ε_*n*_ is the spatial extent of the *n* orbital. With larger orbital lobes that come when stepping down a group in the periodic table (from C to Si, or from N to P), comes a reduction in the intraorbital Coulombic two-electron repulsion. This effect was earlier found to be the dominant factor to explain why parent carbene (CH_2_) has a triplet ground state while parent silylene (SiH_2_) has a closed-shell singlet ground state with two electrons in the *n* orbital.^66^ The species investigated herein all have analogous closed-shell singlet ground states, and thus, the excitation to ^3^ *n*,π* provides for an electrostatic relief. This relief, however, is larger in carbene **9** than in silylene **17** due to the smaller spatial size of the lone-pair orbital at C than at Si.

As the fifth factor, we list the (anti)­aromatic character of the heteroaromatics in its S_0_ and ^3^ *n*,π* states. For the 5-MR species, excluding the mesoionic ones, we saw a correlation between the difference in (anti)­aromatic character between the states, ΔMCI­(S_0_–^3^ *n*,π*), and *E*(^3^ *n*,π*). When the difference in aromaticity between the two states is small, the *E*(^3^ *n*,π*) is low and it is probable that the ^3^ *n*,π* state is lowest among the excited states. The opposite applies when the (anti)­aromaticity difference between the states is large. However, for the order between the ^3^ *n*,π* and ^3^π,π* states, the stabilizing and destabilizing features of the π,π* states must also be taken into consideration. Indeed, there is no correlation between the Δ*E*(^3^π,π*–^3^ *n*,π*) gap and the MCI value of only the ^3^ *n*,π* state (MCI­(^3^ *n*,π*)) of the 5-MR species.

The sixth and final factor that we discuss is the effect of the electronegativities of the atoms adjacent to the E atom. The electronegativities of these atoms also impact strongly on the energy of the ^3^ *n*,π* state as becomes apparent through a comparison of **2** and **4**. The more electronegative N atom adjacent to the carbene center lowers the ε_*n*_ (Figure 8B) by a stronger inductive electron withdrawal than what a sp^2^ hybridized C atom does.

Based on the above, the 7-MR heteroaromatics are more likely to have *n*,π* states as their lowest excited states, as seen through the *C*_2v_ symmetric structure of **24** which has a very low *E*(^3^ *n*,π*). Indeed, by comparison of the *E*(^3^ *n*,π*) and *E*(^3^π,π*) of 6-MR pyridine (**1**) and phosphinine (**26**) with those of 7-MR azepinium cation (**27**) and phosphepinium cation (**28**) one sees that the ^3^ *n*,π* state is lowered in energy relative to the ^3^π,π* state for the larger rings, both when E = N and when E = P (Figure 9A). In summary, by utilizing the various factors listed here it is possible to rationally tune the order between *n*,π* and π,π* states of monocyclic heteroaromatics (Figure 9B).

**Figure 9:**
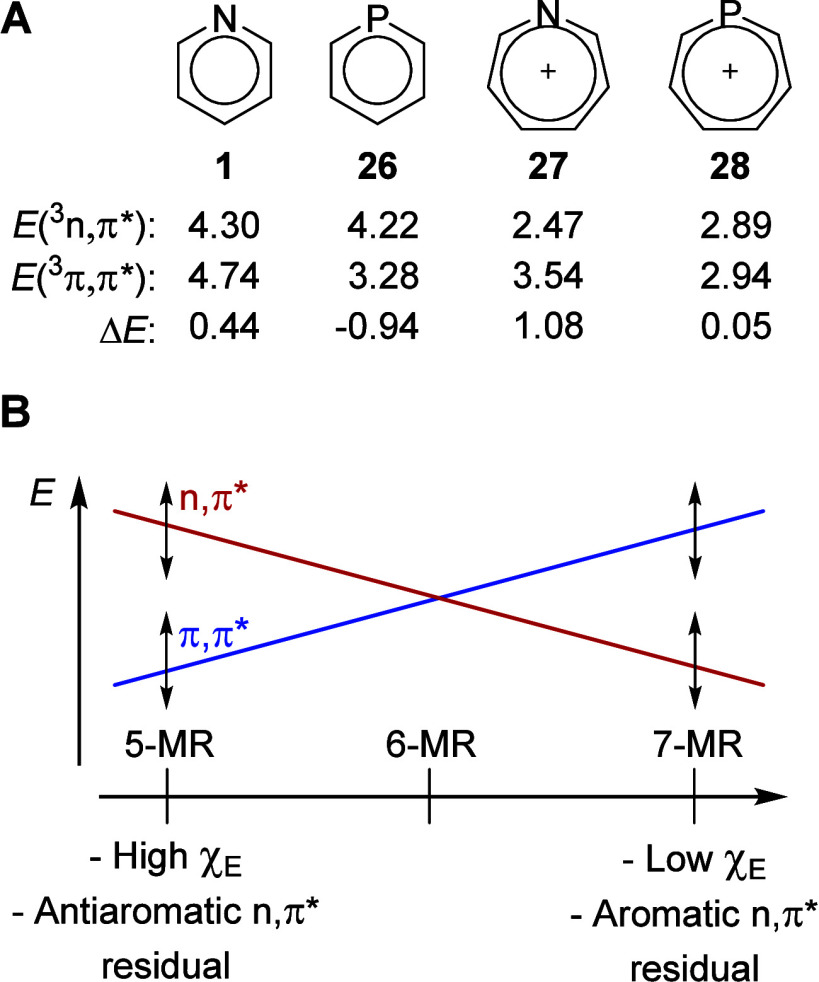
(A) 6-MR pyridine (**1**) and phosphinine (**26**), the 7-MR azepinium and phosphepinium cations (**27** and **28**), and their computed vertical excitation energies to the lowest ^3^ *n*,π* and ^3^π,π* states. (B) Schematic illustrating how the order between the *n*,π* and π,π* states depends on the ring size as well as additional factors, e.g., the electronegativity of E (χ_E_) and the (anti)­aromatic character of the residual of the *n*,π* state, indicated by the double-headed arrows.

## Conclusions and Outlook

Herein, we have taken steps to the development of a systematics for the prediction of which heteroaromatics have *n*,π* states as their lowest excited states and which ones have π,π* states as these. The factors that influence the relative order between the two states are (i) the electronegativity of the heteroatom, (ii) the number of π-orbitals and π-electrons of the ring, (iii) the valence angle at the E atom, (iv) the period to which the heteroelement belongs (second vs third) and thus, the spatial extent of the orbital, (v) the extents of (anti)­aromaticity in the various states, and (vi) the electronegativities of the atoms adjacent to heteroatom E. The aromaticity gap between S_0_ and ^3^ *n*,π* state as determined by MCI correlates well with the vertical energy difference between these states. Based on the six factors we have clarified that 5-MR *N*-heterocyclic carbenes, viewed as 5-MR heteroaromatics, will have *n*,π* states as their lowest excited states. This is in contrast to regular heteroaromatics with one or two N, O, and/or S atoms which never have *n*,π* states as their T_1_ and S_1_ states.

As the ring-size increases, the *E*(*n*,π*) will become lower compared to that of *E*(π,π*), and this is also the case when the electronegativity of the heteroatom E decreases, yet, further investigations are needed. One apparent next step is directed to the π,π* states of heteroaromatics of different ring sizes and their extent of Baird-antiaromatic character. Another next step is to explore the *n*,π* and π,π* states of heteroaromatics with three or more heteroatoms, and a third is to explore the geometrically relaxed excited states more comprehensively, especially the singlet excited states. The information from a final and complete systematics that could be derived can tentatively be useful input information for machine learning on various excited state properties of molecules with heteroaromatic cycles, e.g., the optical properties of larger polycyclic compounds (partially) with heteroaromatic cycles.

## Computational Methods

### Optimizations and Energy Calculations

All geometry optimizations and energy calculations of the compounds of Figure 1 were performed with Gaussian 16 revision B.01,^67^ using the CAM-B3LYP^68^ functional and the 6-311+G­(d,p) basis set.^69^ For the calculations of energies and wave functions in the subsequent aromaticity investigations, 6d and 10f Cartesian functions were added to this basis set. This level of theory was selected based on benchmark calculations performed in our previous work.^13^ Single-reference methods like unrestricted DFT are particularly well suited for calculating the lowest triplet excited states, especially those of *n*,π* character (see ref 70). Restricted Kohn–Sham DFT was used for optimizations of the S_0_ state closed-shell structures, while the unrestricted DFT formalism was applied to obtain energies and properties of the triplet states. In the cases where the ^3^ *n*,π* state was not the lowest triplet, the orbital order was changed using the guess = alter keyword in Gaussian. Other triplet states with ^3^ *n*,π* and ^3^π,π* character were also explored using this approach. Time-dependent DFT (TD-DFT) was used to obtain the energies of vertically excited states with singlet multiplicity. All calculations were performed using symmetry and quadratic convergence for the SCF. The stability of the wave functions were analyzed for all the S_0_ and ^3^ *n*,π* states. In most of the cases, the lowest triplet state has ^3^ *n*,π* character and the wave function is stable. In the cases where the ^3^ *n*,π* state was not the lowest excited state, the orbital order had to be altered and the resulting wave function was found unstable. Optimization of this wave function forces the species back to the lower ^3^π,π* state, which leads us to conclude that the wave functions of these ^3^ *n*,π* states are stable within the considered symmetry.

To ensure that the UDFT wave function can be properly used for computing electronic-based indices for aromaticity for the states of interest, we evaluate the multiconfigurational character of selected compounds using CASSCF and their energies with CCSD, similarly to our previous work.^13^ It is well-stablished that CASSCF wave functions provide inaccurate estimations of the energy gap between *n*,π* and π,π* states and also provide MCI values that are always lower than those obtained at the DFT level (see refs 24 and 71). Usually, the trends provided by the DFT and CASSCF methods are the same, except in cases where single-reference methods provide an inaccurate description of the wave function. CCSD was used as a secondary reference to access the quality of the (U)­DFT wave functions, although this method is often more accurate for π,π* than *n*,π* states. However, our primary goal in this work is not to accurately determine the energetic difference between these states but rather to evaluate the aromatic character of the *n*,π* states. Therefore, such uncertainties do not significantly affect the observed trends.

Additionally, for selected compounds we performed DFT/MRCI (combined density functional theory and multireference configuration interaction)^72,73^ calculations, with the integrals generated by Turbomole v.7.5.^74,75^ Symmetry was kept in all calculations. The R2018 redesigned Hamiltonian^85^ with a threshold parameter of 1.0 hartree and aug-cc-pVTZ basis set was employed. We found for a set of selected compounds (Table S15) that the lowest ^3^ *n*,π* states are properly characterized as singly excited states, as the percentage of double excitation is smaller than 10%, with one exception (compound **4**, which has 11%).

### Aromaticity Assessments

The aromaticity was assessed by using different indices based on electronic and magnetic criteria, with separation of the α and β spins. The multicenter index (MCI) method,^44,45^ which indicates the electron delocalization between different atoms of a molecule, was performed at (U)­CAM-B3LYP/6-311+G­(d,p) level of theory using the AIMAll^76^ and ESI-3D^24,77^ packages. With the MCI index, the threshold for a molecule to be labeled as aromatic in the S_0_ state varies with ring size. As reference MCI values for maximally aromatic species we use benzene, cyclopentadienyl anion, and cyclopropenium cation in S_0_, and as a general threshold for aromaticity we use half of the values of these species. The residual in the *n*,π* state is expressed as the percentage of retained S_0_ aromaticity. With MCI, we consider a value at 50% to indicate nonaromaticity, while a deviation from this of 10% in either direction corresponds to a residual of aromatic or antiaromatic character.

Magnetic properties were evaluated with magnetically induced current densities (MICDs) calculated using the CTOCD-DZ (continuous transformation of origin of current density–diamagnetic zero)^46–49^ method at CAM-B3LYP/6-311+G­(d,p) level of computations. A perpendicular external magnetic field was applied to the molecular plane. The maps of current density were generated at a distance of 1 bohr above the ring plane. Clockwise and counterclockwise circulations represent diatropic and paratropic current densities, respectively. Bond current strengths^78^ were obtained by numerically integrating the current densities that pass through a rectangle dividing the bond center. This integration rectangle originated from the ring center and extended 5 bohr away from it, beyond the molecular ring. The rectangle covered an area of 5 bohr above and below the ring plane. The ring current strengths were computed as the mean current strengths of all bonds within a given ring.

The energetic aspect of (anti)­aromaticity was evaluated in selected cases through the isomerization stabilization energy (ISE) approach, where the energy of a methyl substituted (hetero)­aromatic molecule is compared to that of a methylene-substituted isomer with a linear π-conjugated segment.^54^ Significantly negative ISE values indicate aromatic stabilization, positive values antiaromatic destabilization and values close to zero nonaromatic character. Noteworthy, the ISE values are computed at optimized (relaxed) geometries on the S_0_ and ^3^ *n*,π* states. These calculations were done at (U)­CAM-B3LYP/6-311+G­(d,p) level of theory.

Finally, a limited number of compounds at their S_0_ and relaxed ^3^ *n*,π* state geometries were examined using the HOMA (harmonic oscillator model of aromaticity) geometric descriptor of (anti)­aromaticity, where values approaching 1.0 (from below) indicate aromaticity and values below zero antiaromaticity.^55^

## Supplementary Material



## Data Availability

The data underlying this study are openly available in the published article and its Supporting Information and also openly available in ioChem-BD at 10.19061/iochem-bd-4-84

## Notes

Further analysis of the 3-MRs; aromaticity data (MCI, MICD, HOMA, EDDB); further analysis of excited-state character; energies; TD-DFT results; orbital diagrams; DFT/MRCI calculations; solvent effects; charge analysis; angle dependency; correlation plots; and Cartesian coordinates, total energies, and number of imaginary frequencies (PDF)
