# Supplementary material for: Toward a Systematics for the Lowest Excited States of Heteroaromatics Enabled via Cyclic π‑Conjugated Carbenes and Heteroelement Analogues
Source: J Org Chem. 2025 Jul 8;90(28):9743–56. doi: 10.1021/acs.joc.5c00578 (PMC12281578; doi:10.1021/acs.joc.5c00578)
Supplement: Supplementary file 1 [file jo5c00578_si_001.pdf]

SUPPORTING INFORMATION

Toward a Systematics for the Lowest Excited States of Heteroaromatics Enabled *via* Cyclic π-Conjugated Carbenes and Heteroelement Analogues

Nathalie Proos Vedin,[a] Josene M. Toldo,[b] Sílvia Escayola,[c,d,e] Slavko Radenković,[f]*

Miquel Solà[c]* and Henrik Ottosson[a]*

[a] Department of Chemistry - Ångström, Uppsala University, 751 20 Uppsala, Sweden.

[b] Université Claude Bernard Lyon 1, Laboratoire de Chimie UMR 5182, ENS de Lyon, CNRS, 69342, Lyon cedex 07, France. [c] Institut de Química Computacional i Catàlisi and Departament de Química, Universitat de Girona, C/ Maria Aurèlia Capmany, 69, 17003 Girona, Catalonia, Spain. [d] Donostia International Physics Center (DIPC), 20018 Donostia, Euskadi, Spain. [e] Institute for Theoretical Chemistry, University of Stuttgart, Pfaffenwaldring 55, 70569 Stuttgart, Germany. [f] University of Kragujevac, Faculty of Science, P. O. Box 60, 34000 Kragujevac, Serbia.

Table of Contents

1 3-MRs: Further Analysis S3
2 Aromaticity Data S6
2.1 MCI S6
2.2 MICD S9
2.3 HOMA S9
2.4 EDDB S10
3 Further Analysis S11
3.1 State Characterization S11
3.2 Energies S12
3.3 TD-DFT of Compound 19 ..... S14
3.4 Orbitals S15
3.5 DFT/MRCI S15
3.6 Solvent Effect S16
3.7 Charges S16
3.8 Angle Dependency S17
3.9 Correlations S17
4 Coordinates, total energies & number of imaginary frequencies S20
References S28

1 3-MRs: Further Analysis

As described in the main text, the last class of compounds explored were the 3-MR cyclopropenylidene (**19**), the isoelectronic B^- and N^+ analogues **20** and **21**, and the heavier Si and P^+ analogues **22** and **23**. Firstly, we again notice that the B-containing compound **20** in the gas phase has a triplet state of n,Ry character below its lowest n,π* state, similar to **5** and **15**, but it moves up in energy when the molecule is embedded in a solvent cavity simulated by a polarizable continuum model. Also, the lowest excited states of **22** and **23** are of ^{3}n,π* character. The S_1 states of **19** – **21** are all of n,π* character, but for **19** it is of different state symmetry than the T_1 state (A_2 instead of B_1 symmetry). On the other hand, compounds **22** and **23** have S_1 states of σ,π* character below their lowest ^{1}n,π* states.

With regard to the relatively high MCI value of the lowest ^{3}n,π* state of **19** (80% when compared to the value of the S_0 state), it is especially noteworthy that the MCI_α-component is large, as this suggests that the $2\pi_\alpha$-electron component is able to alleviate antiaromaticity efficiently without geometric relaxation. Conversely, the MCI_β-component is just slightly stronger than in S_0. Combined, this leads to a residual in the ^{3}n,π* state of **19** which strongly leans towards aromaticity. Now what is (are) the cause(s) of this large MCI residuals in the vertical ^{3}n,π* state; are they necessarily caused by aromaticity? There can be three causes. First, by dissecting the σ- and π-contributions to the MCI_α component of **19** (in both S_0 and ^{3}n,π*) it becomes clear that the σ-contributions are substantial, leading to a higher MCI_α value than in the larger cycles. This indicates σ-aromaticity, a form of aromaticity that exists in, *e.g.*, H_3^+,[1-3] even though it was concluded to have minor significance in cyclopropane.[4] When the σ-contributions are excluded, the residual based on MCI is, however, still above what is required to be categorized as aromatic (Table S5). A second factor is that the n,π* transition in the 3-MRs is an excitation of one electron into an electron-deficient π-orbital scaffold which has two π-electrons on three atoms. This means that the excitation attenuates the Coulombic two-electron repulsion within the n orbital at the cost of a slight increase in the electron repulsion within the π-orbitals. A third factor that influences the residual is the C_{2v} symmetric S_0 geometry with markedly different C-C bond lengths (1.319 and

1.410 Å, Figure S1A). Thereby, the antiaromatic character becomes alleviated without any further geometric distortion as the S_0 geometry already accommodates the required relaxation.

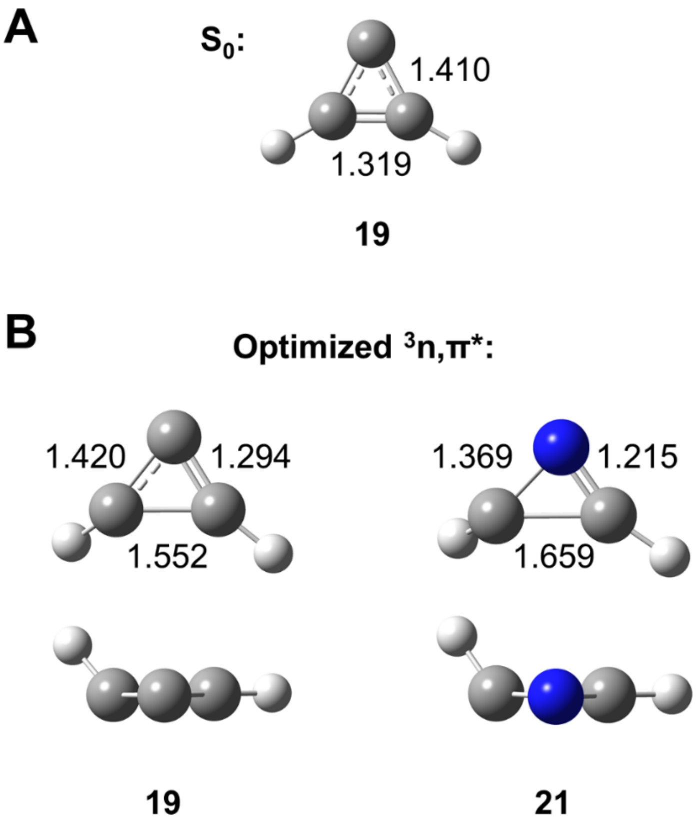

Figure S1. Optimized geometries of (A) compound **19** in S_0 and (B) the lowest ^{3}n,π* state, as well as of compound **21** in the ^{3}n,π* state, calculated at CAM-B3LYP/6-311+G(d,p) level of theory.

Interestingly, in their vertical ^{3}n,π* states both **20** and **21** have slightly more aromatic character according to MCI than what is the case in S_0, which is different from **19** which becomes less aromatic in ^{3}n,π* than in S_0. As **21** was already strongly aromatic in S_0, one may ask how come **21** is so strongly aromatic in its vertical ^{3}n,π* state? When compared to **19**, compound **21**, composed of atoms with different electronegativities, could have an even better ability to host the two π_α-electrons and simultaneously alleviate the π_α-antiaromaticity. In support of this, the MCI_α contribution has a slightly higher value than in the ^{3}n,π* state of **19**. Despite this, the ^{3}n,π* state

of **21** when relaxed geometrically distorts to a nonplanar structure with one H atom out of plane (Figure S1B), resembling the optimized geometry of **19**.

As briefly mentioned in the main text, the emergence of what has been labelled as adaptive aromaticity is not a new and unique form of aromaticity, since the aromatic character of the open-shell state can be rationalized through Mandado's rule. As an illustration, the spin-separated analysis of the $^3n,\pi^*$ state of **19** reveals that its aromatic character observed in the computed MCI data of the T_1 state is due to an insufficient cancellation between the aromatic MCI_β component and antiaromatic MCI_α component. This resembles what we earlier found for some regular heteroaromatics, *e.g.*, pyrazine in its $^3n,\pi^*$ state.[5] In addition, in **19** there is the potential σ-electron contribution to the residual. Combined, this highlights the fact that computations alone are insufficient for analysis of excited state (anti)aromaticity effects; qualitative electronic structure considerations are required for comprehensive analyses. Thus, to rely solely on computational observations can be misleading, the computed values must be analysed and rationalized chemically.

At this point, one may wonder if the markedly aromatic residual of the vertically excited n,π^* state is reflected in the photochemistry of **19**. To assess this, we considered the lowest singlet n,π^* states, and the MCI values of these resemble those of the triplet states of both B_1 and A_2 symmetries (Table S6). Yet, despite the fact that both triplet and singlet n,π^* states have residuals corresponding to considerable aromaticity, geometric relaxation leads to highly distorted nonplanar structures with three unequal CC bonds and one H atom out-of-plane (Figure S1B). This should not be surprising as the $^3n,\pi^*$ state has two π_α- and one π_β-electron, and the two π_α-electrons are distributed on only three atoms. Clearly, the Pauli repulsion is stronger than the aromatic stabilization of the planar structure, and thereby the molecule distorts from planarity. Noteworthy, a combined time-resolved photoelectron spectroscopic and quantum dynamics study found the nonplanar S_1 structure of **19** to be connected to several conical intersections between S_1 and S_0 leading to ring-opened structures and photodegradation.[6]

2 Aromaticity Data

2.1 MCI

Table S1. MCI results of the S_0 and the lowest vertical $^3n,\pi^*$ state of all compounds of Figure 1, calculated at CAM-B3LYP/6-311+G(d,p) level of theory. The percentages are based on absolute values.

	S_0		Lowest vertical $^3n,\pi^*$						
**Compound**	**MCI**	**50% of MCI(S_0)**	**MCI**	**% of MCI(S_0)**	**MCI_α**	**% of MCI(S_0)**	**MCI_β**	**% of MCI(S_0)**	**Symm**
**1**	0.0674	0.0337	0.0304	45	0.0028	4	0.0276	41	B_1
**2**	0.0391	0.0195	0.0247	63	0.0020	5	0.0227	58	A″
**3**	0.0322	0.0161	0.0258	80	0.0027	8	0.0231	72	A″
**4**	0.0227	0.0113	0.0245	108	0.0027	12	0.0218	96	B_1
**5**	-0.0005	0.0002	0.0049	998	0.0017	346	0.0032	652	B_1
**6**	0.0462	0.0231	0.0298	65	0.0026	6	0.0272	59	B_1
**7**	0.0062	0.0031	0.0073	118	0.0011	17	0.0062	101	A″
**8**	0.0392	0.0196	0.0272	69	0.0013	3	0.0259	66	A″
**9**	0.0389	0.0195	0.0229	59	0.0004	1	0.0225	58	B_1
**10**	0.0433	0.0217	0.0235	54	0.0007	2	0.0228	53	A″
**11**	0.0378	0.0189	0.0228	60	0.0001	0	0.0227	60	A″
**12**	0.0468	0.0234	0.0314	67	0.0002	1	0.0312	67	A″
**13**	0.0483	0.0241	0.0281	58	0.0008	2	0.0274	57	B_1
**14**	0.0570	0.0285	0.0329	58	0.0009	2	0.0320	56	A″
**15**	0.0202	0.0101	0.0220	109	0.0105	52	0.0115	57	B_1
**16**	0.0625	0.0313	0.0315	50	0.0002	0	0.0313	50	B_1
**17**	0.0272	0.0136	0.0159	59	0.0014	5	0.0145	53	B_1
**18**	0.0446	0.0223	0.0262	59	0.0008	2	0.0254	57	B_1
**19**	0.3761	0.1880	0.3001	80	0.0924	25	0.2078	55	B_1
**20**	0.1859	0.0930	0.2030	109	0.0920	50	0.1110	60	B_1
**21**	0.3634	0.1817	0.3718	102	0.1014	28	0.2704	74	B_1
**22**	0.2083	0.1041	0.1840	88	0.0633	30	0.1207	58	B_1
**23**	0.3299	0.1650	0.2556	77	0.0739	22	0.1817	55	B_1

Table S2. MCI results of the S_0 and the lowest vertical $^3n,\pi^*$ state of selected compounds, calculated at CCSD/6-311+G(d,p) level of theory.

	**S_0**		**Lowest vertical $^3n,\pi^*$**						
**Compound**	**MCI**	**50% of MCI(S_0)**	**MCI**	**% of MCI(S_0)**	**MCI_α**	**% of MCI(S_0)**	**MCI_β**	**% of MCI(S_0)**	**Symm**
**1**	0.0443	0.0222	0.0206	47	0.0021	5	0.0185	42	B_1
**2**	0.0242	0.0121	0.0153	63	0.0013	6	0.0140	58	A″
**3**	0.0203	0.0101	0.0160	79	0.0020	10	0.0141	69	A″
**4**	0.0137	0.0069	0.0149	108	0.0019	14	0.0130	94	B_1
**9**	0.0259	0.0129	0.0145	56	0.0001	0	0.0144	56	B_1
**10**	0.0291	0.0145	0.0152	52	0.0003	1	0.0148	51	A″
**11**	0.0248	0.0124	0.0147	59	0.0000	0	0.0148	60	A″
**12**	0.0311	0.0155	0.0212	68	0.0000	0	0.0212	68	A″
**14**	0.0382	0.0191	0.0216	57	0.0004	1	0.0212	55	A″
**17**	0.0173	0.0086	0.0093	54	0.0009	5	0.0084	49	B_1
**18**	0.0293	0.0147	0.0170	58	0.0004	2	0.0166	56	B_1
**19**	0.3255	0.1627	0.2753	85	0.0681	21	0.2072	64	B_1
**22**	0.1722	0.0861	0.1527	89	0.0571	33	0.0955	55	B_1

Table S3. MCI results of the S_0 and the lowest vertical $^3n,\pi^*$ state of selected compounds, calculated at B3LYP/6-311+G(d,p) level of theory.

	**S_0**		**Lowest vertical $^3n,\pi^*$**						
**Compound**	**MCI**	**50% of MCI(S_0)**	**MCI**	**% of MCI(S_0)**	**MCI_α**	**% of MCI(S_0)**	**MCI_β**	**% of MCI(S_0)**	**Symm**
**1**	0.0679	0.0340	0.0314	46	0.0028	4	0.0286	42	B_1
**2**	0.0410	0.0205	0.0259	63	0.0021	5	0.0238	58	A″
**9**	0.0408	0.0204	0.0242	59	0.0006	2	0.0235	58	B_1
**17**	0.0303	0.0152	0.0179	59	0.0017	5	0.0163	54	B_1
**18**	0.0457	0.0229	0.0270	59	0.0010	2	0.0260	57	B_1
**19**	0.3783	0.1891	0.3234	85	0.0724	19	0.2510	66	B_1
**22**	0.2159	0.1079	0.1873	87	0.0635	29	0.1238	57	B_1

Table S4. MCI results of the S_0 and the lowest vertical ^{3}n,π* state of selected compounds, calculated at BLYP/6-311+G(d,p) level of theory.

	S_0		Lowest vertical ^{3}n,π*						
**Compound**	**MCI**	**50% of MCI(S_0)**	**MCI**	**% of MCI(S_0)**	**MCI_α**	**% of MCI(S_0)**	**MCI_β**	**% of MCI(S_0)**	**Symm**
**1**	0.0704	0.0352	0.0335	48	0.0025	4	0.0310	44	B_1
**2**	0.0449	0.0225	0.0280	62	0.0021	5	0.0259	58	A″
**9**	0.0451	0.0225	0.0268	60	0.0011	2	0.0258	57	B_1
**17**	0.0346	0.0173	0.0209	60	0.0020	6	0.0188	54	B_1
**18**	0.0493	0.0247	0.0297	60	0.0014	3	0.0283	57	B_1
**19**	0.3812	0.1906	0.3075	81	0.0925	24	0.2150	56	B_1
**22**	0.2247	0.1123	0.1896	84	0.0632	28	0.1265	56	B_1

Table S5. MCI results, dissected into σ- and π-contributions**,** of the S_0 and the lowest vertical ^{3}n,π* state of compounds **19**, calculated at CAM-B3LYP/6-311+G(d,p) level of theory.

	S_0		Lowest vertical ^{3}n,π*					
**Compound**	**MCI**	**50% of MCI(S_0)**	**MCI**	**% of MCI(S_0)**	**MCI_α**	**% of MCI(S_0)**	**MCI_β**	**% of MCI(S_0)**
**Total**	0.3761	0.1880	0.3001	80	0.0924	25	0.2078	55
**σ**	0.1239	0.0620	0.1437	116	0.0593	48	0.0844	68
**π**	0.2521	0.1261	0.1564	62	0.0330	13	0.1234	49

Table S6. MCI results for the lowest singlet and triplet excited states of compound **19**, both of n,π* character, calculated at TD-CAM-B3LYP/6-311+G(d,p) level of theory.

State	Symm	MCI
**T_1**	B_1	0.2278
**T_2**	A_2	0.2844
**S_1**	A_2	0.2830
**S_2**	B_1	0.2517

2.2 MICD

Table S7. Total and π-electron ring current strengths (in nA T^{-1}) calculated as the average of all bonds in the given ring. ΔE_{H-L} (in Hartrees) is the HOMO-LUMO energy gap for π_α-orbitals.

	Comp.	S0				3n,π*						
		π	$\pi_{\alpha/\beta}$	total	$total_{\alpha/\beta}$	π	π_α	π_β	total	$total_\alpha$	$total_\beta$	ΔE_{H-L}
**6MR**	**1**	11.1	5.5	11.2	5.6	-19.8	-24.9	5.1	-19.9	-25.5	5.6	
	**4**	9.4	4.7	9.3	4.7	-11.0	-15.7	4.7	-11.3	-16.1	4.8	
	**5**	6.5	3.3	6.3	3.2	-18.3	-21.5	3.3	-19.0	-22.2	3.2	
	**6**	10.4	5.2	10.5	5.2	-6.6	-11.9	5.3	-6.5	-12.2	5.7	
	**Benzene**	**11.5**	**5.8**	**11.8**	**5.9**							
**5MR**	**9**	7.5	3.7	9.7	4.8	-5.3	-9.2	3.8	-4.6	-8.4	3.8	0.196
	**13**	7.0	3.5	11.5	5.7	-11.2	-14.8	3.6	-11.6	-13.1	1.5	0.193
	**14**	8.1	4.1	12.3	6.2	-7.6	-11.7	4.1	-7.8	-10.2	2.4	0.203
	**16**	9.0	4.5	11.0	5.5	0.5	-4.1	4.7	0.4	-3.4	3.7	0.273
	**Cp^-**	**11.1**	**5.6**	**12.5**	**6.2**							
**3MR**	**19**	3.8	1.9	11.1	5.5	-16.7	-18.5	1.9	-15.4	-15.3	-0.1	0.194
	**20**	3.4	1.7	8.9	4.5	-23.4	-25.0	1.7	-21.8	-22.5	0.7	0.123
	**21**	3.7	1.9	10.7	5.4	-6.5	-8.4	1.9	-5.3	-5.1	-0.2	0.247

2.3 HOMA

Table S8. HOMA values for a few of the 6-MRs in both the S_0 and lowest ^{3}n,π* states, as well as HOMER values for the latter state. It should be noted that an excited state of n,π* character could be considered as a mix between a closed-shell singlet ground state and an excited π,π* state, and that neither of these indices have been parametrized with such a situation in mind.

Compound	S0	3n,π*	
	**HOMA**	**HOMA**	**HOMER**
**4**	0.8520	0.7961	-1.2880
**9**	0.7791	0.4281	-1.0604
**14**	0.9180	0.2291	-1.0881

2.4 EDDB

In the main text, we report ISE values that indicate clear aromatic character in S_0 for compound **4**. However, when looking more closely at the optimal bond lengths and the electron delocalization of the nonaromatic isomers one can note that the linear π-conjugated triene segments are best described as one 1,3-diene unit plus one ethylene segment separated by a CC single bond (Figure S2). This indicates that the nonaromatic isomers used in our ISE reactions do not represent a fully π-conjugated linear 1,3,5-triene segment, and as a consequence, they are of too high relative energies. Thus, the computed ISE values are more negative than if they exclusively had represented the aromatic stabilization energy.

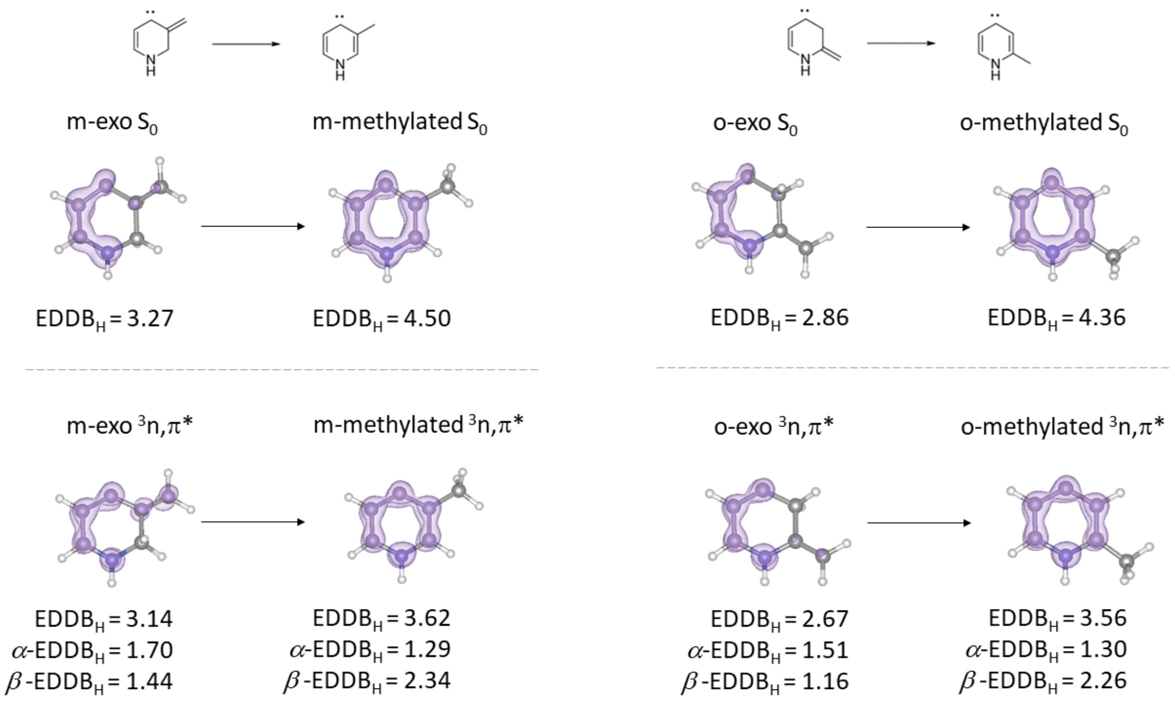

Figure S2. $EDDB_H$ (electron density of delocalized bonds) surfaces and values for species involved in the ISE reactions of compound **4** in the S_0 and lowest $^3n,\pi^*$ states. In the latter case, the $EDDB_H$ values are dissected into α- and β-components. The calculations were done at CAM-B3LYP/6-311+G(d,p) level of theory and the units for $EDDB_H$ are electrons.

3 Further Analysis

3.1 State Characterization

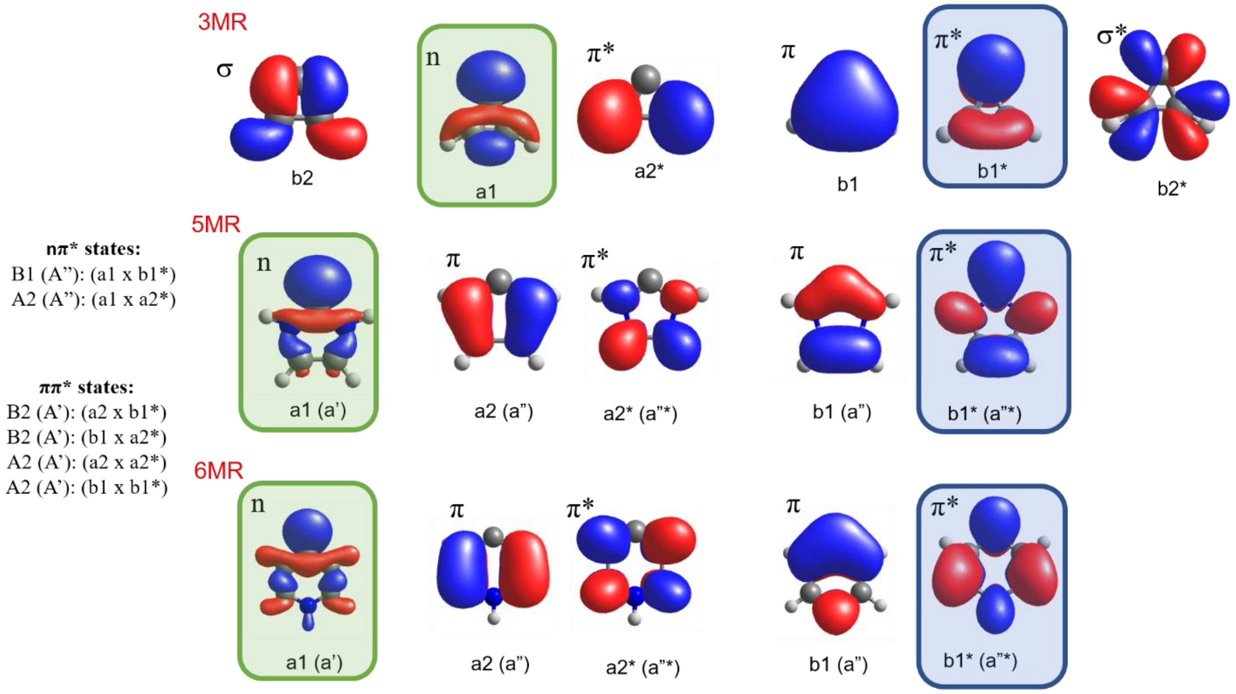

Figure S3. Molecular orbitals and state symmetries.

3.2 Energies

Table S9. Absolute energies of the S_0 and the lowest vertical $^3n,\pi^*$ state of all compounds of Figure 1, calculated at CAM-B3LYP/6-311+G(d,p) level of theory. The vertical excitation energies are given in Table 1.

	**Absolute Energy (Ha)**	
**Compound**	**S_0**	**Lowest $^3n,\pi^*$**
**1**	-248.21310812	-248.05501668
**2**	-248.14421404	-248.06256331
**3**	-248.11884190	-248.06125350
**4**	-248.11745447	-248.07393214
**5**	-234.87291399	-234.85458326
**6**	-264.59475815	-264.48534056
**7**	-499.57994852	-499.49759119
**8**	-551.22573134	-551.10286070
**9**	-226.13088306	-225.97403701
**10**	-226.09775317	-225.96347554
**11**	-242.17173725	-242.01262357
**12**	-242.11857444	-241.99564543
**13**	-258.18651581	-258.02208172
**14**	-258.15330398	-258.03074625
**15**	-212.91758787	-212.83879052
**16**	-242.53311352	-242.31449032
**17**	-477.61717279	-477.47283086
**18**	-529.22143805	-529.02245836
**19**	-115.31808458	-115.20252070
**20**	-102.09934294	-102.02387317
**21**	-131.70316894	-131.55850186
**22**	-366.80000586	-366.68376103
**23**	-418.38172075	-418.24512153

Table S10. Absolute energies of the S_0 and the lowest vertical $^3n,\pi^*$ and $^3\pi,\pi^*$ state of the additional compounds presented in Figures 8 and 9, calculated at CAM-B3LYP/6-311+G(d,p) level of theory.

	**Absolute Energy (Ha)**		
**Compound**	**S_0**	**Lowest $^3n,\pi^*$**	**Lowest $^3\pi,\pi^*$**
**24**	-270.1139934	-270.0975495	-269.9954363
**25**	-140.7137394	-140.7146607	-140.5559407
**26**	-534.82546526	-534.67031797	-534.704767781
**27**	-286.58844588	-286,49784826	-286,458261034
**28**	-573,21249019	-573,10641527	-573,104490087

Table S11. Absolute energies of the two lowest vertical $^3n,\pi^*$ states for a few of the 3-MRs, calculated at CAM-B3LYP/6-311+G(d,p) level of theory.

	**Absolute Energy (Ha)**		**Energy difference (eV)**
**Compound**	**T_1 ($^3B_1$)**	**T_2 ($^3A_2$)**	**T_2 ($^3A_2$) – T_1 ($^3B_1$)**
**19**	-115.2025207	-115.1796104	0.62
**22**	-366.6837610	-366.6268288	1.55
**23**	-418.2451215	-418.1676167	2.11

Table S12. Absolute energies of the S_0 and the lowest vertical $^3n,\pi^*$ state of selected compounds, as well as the vertical excitation energies and the T_1 diagnostics values, calculated at CCSD/6-311+G(d,p) level of theory.

	**Absolute Energy (Ha)**		**Vertical Excitation Energy (eV)**	**T_1 diagnostics**	
**Compound**	**S_0**	**Lowest $^3n,\pi^*$**	**Lowest $^3n,\pi^*$**	**S_0**	**Lowest $^3n,\pi^*$**
**1**	-247.6443945	-247.4803812	4.46	0.0125	0.0397
**2**	-247.5757304	-247.4910769	2.30	0.0158	0.0395
**3**	-247.5479984	-247.4887599	1.61	0.0136	0.0298
**4**	-247.5470402	-247.5039100	1.17	0.0163	0.0348
**9**	-225.6254602	-225.4670121	4.31	0.0163	0.0171

**10**	-225.5912466	-225.4520665	3.79	0.0158	0.0399
**11**	-241.6435802	-241.4826858	4.38	0.0174	0.0316
**12**	-241.5887649	-241.4612231	3.47	0.0133	0.0413
**14**	-257.6017274	-257.4722041	3.52	0.0163	0.0480
**17**	-476.7215196	-476.5781338	3.90	0.0164	0.0170
**18**	-528.3084125	-528.1060203	5.51	0.0133	0.0172
**19**	-115.0536093	-114.9112148	3.87	0.0115	0.0326
**22**	-366.1452151	-366.0323682	3.07	0.0147	0.0205

3.3 TD-DFT of Compound 19

Table S13. Energies of the lowest excited states of states of compound **19**, calculated at TD-CAM-B3LYP/6-311+G(d,p) level of theory.

Compound	State	Type	Symm	Vertical Excitation Energy (ev)
**19**	T_1	n,π*	B_1	2.89
	T_2	n,π*	A_2	3.78
	S_1	n,π*	A_2	4.04
	S_2	n,π*	B_1	4.78

Table S14. The character and symmetry of the five lowest vertically excited states of **19**, of both singlet and triplet multiplicity, calculated at TD-CAM-B3LYP/6-311++G(2df,2dp) level of theory (this is a different basis set that the rest of the results presented herein).

Compound	State	Type	Symm
**19**	T_1	n,π*	B_1
	T_2	n,π*	A_2
	T_3	σ,π*	B_1
	T_4	σ,π*	A_2
	T_5	n,σ*	A_1
	S_1	n,π*	A_2
	S_2	n,π*	B_1
	S_3	n,σ*	A_1
	S_4	σ,π*	B_1
	S_5	n,σ*	B_2

3.4 Orbitals

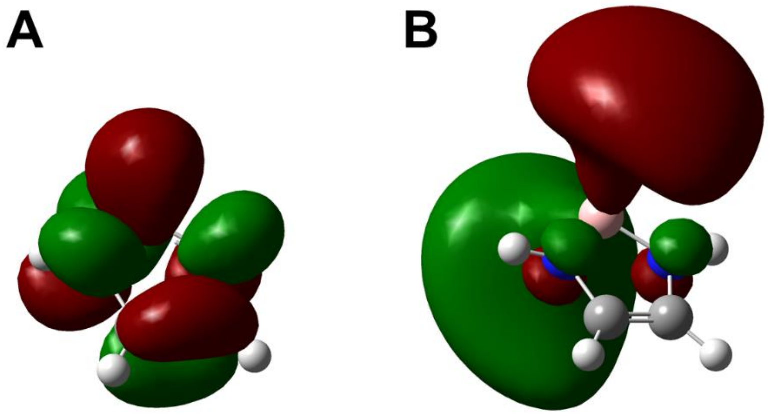

Figure S4. The π* orbitals that become occupied upon n,π* excitation of (A) compound **9** and (B) compound **15**. The latter is more diffuse and could be classified as a Rydberg orbital, as discussed in the main text.

3.5 DFT/MRCI

Table S15. DFT/MRCI calculations for selected compounds. Percentage of single excitation (%S) and percentage of doubly excitation (%D) in the triplet state of symmetry B_1 (a_1 x b_1, C_{2v} symm) or A'' (a' x a'', C_s symm). %T and %Q are not included.

Compound	%S	%D
**2**	94	5
**4**	88	11
**9**	91	8
**13**	94	6
**14**	90	10
**18**	90	9
**19**	96	4
**20**	92	8

3.6 Solvent Effect

Table S16. Gas phase and PCM calculations for compounds containing boron.

		UDFT			TDDFT			
**Compound**	**Solvent**	***E*(T_1) (eV)**	**T_1 Type (symm)**	**^{3}n,π* (B_1) (eV)**	***E*(T_1) (eV)**	**T_1 symm**	***E*(S_1) (eV)**	**S_1 Symm**
**5 (6-MR)**	**Gas**	0.28	n-Ry (A_1)	0.50	0.29	B_1	1.35	B_1
	**THF**	0.94	n,π* (B_1)	0.94	1.11	B_1	1.86	B_1
	**MeOH**	1.06	n,π* (B_1)	1.06	1.31	B_1	1.97	B_1
**15 (5-MR)**	**Gas**	1.64	n-Ry (A_1)	2.14	1.80	A_1	1.99	A_1
	**THF**	2.72	n-Ry (A_1)	2.81	2.96	B_1	3.43	B_1
	**MeOH**	2.80	n-Ry (A_1)	2.89	3.06	B_1	3.49	B_1
**20 (3-MR)**	**Gas**	1.99	n-Ry (A_1)	2.05	1.98	B_1	2.31	A_1
	**THF**	2.41	n,π* (B_1)	2.41	2.36	B_1	3.23	A_2
	**MeOH**	2.45	n,π* (B_1)	2.45	2.44	B_1	3.31	B_1

3.7 Charges

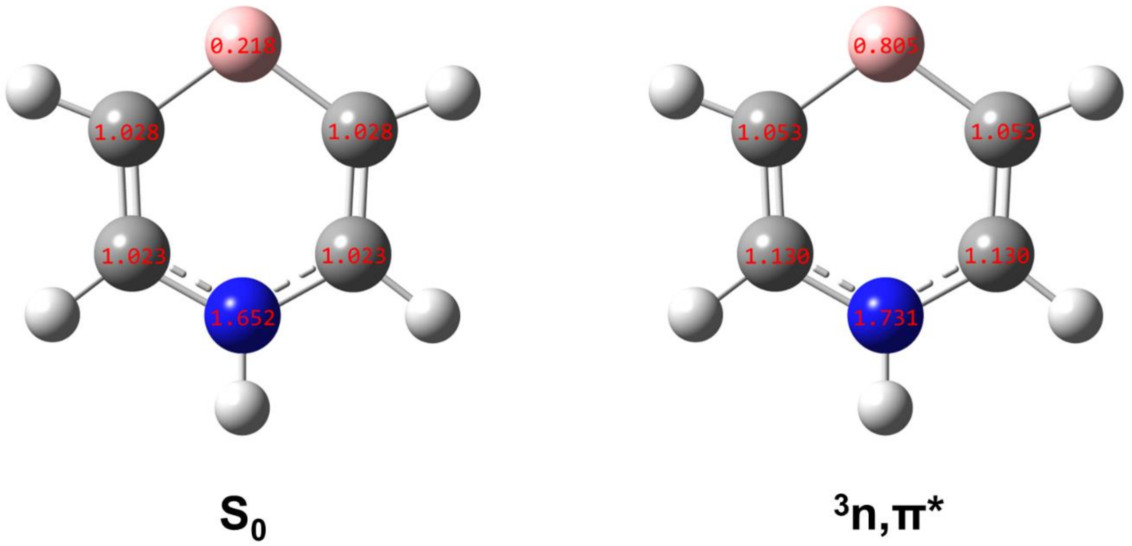

Figure S5. Natural atomic orbital (NAO) populations of the p_z orbitals of compound **5** in S_0 and the lowest vertical ^{3}n,π* state, calculated at CAM-B3LYP/6-311+G(d,p) level of theory.

3.8 Angle Dependency

Table S17. The data that Figure 4 is based on, *i.e.* the energies of the lowest excited states of $H_2N\text{-}C\text{-}NH_2$ at different ∠N-C-N bond angles. The results were obtained at TD-CAM-B3LYP/6-311+G(d,p) level of theory and the values in bold are the ones at the optimized geometry.

Angle (degrees)	Energy (eV)			
	**^{3}n,π***	**^{3}π,π***	**^{1}n,π***	**^{1}π,π***
90	4.13	5.01	6.28	6.39
95	3.34	4.64	5.42	6.03
100	2.93	4.47	4.79	6.00
105	2.39	4.43	4.34	5.98
110	2.13	4.48	4.01	6.06
**112.98**	**2.01**	**4.55**	**3.86**	**6.13**
115	1.95	4.61	3.77	6.27
120	1.85	4.79	3.61	6.45
125	1.82	5.01	3.50	6.69
130	1.83	5.27	3.44	6.91
135	1.89	5.55	3.42	7.46
140	1.98	5.85	3.43	7.73

3.9 Correlations

Table S18. The data that Figure 6 is based on, *i.e.* the energies and MCI values of the regular 5-MR NHCs and their F- and SiH_3-substituted derivatives. The results were obtained at CAM-B3LYP/6-311+G(d,p) level of theory.

	Absolute Energy (Ha)			MCI	
**Compound**	**S_0**	**^{3}n,π***	**^{3}π,π***	**S_0**	**^{3}n,π***
**9**	-226.1308831	-225.9740370	-225.9575975	0.0389	0.0229
**11**	-242.1717373	-242.0126236	-241.9830349	0.0378	0.0228
**13**	-258.1865158	-258.0220817	-257.9955701	0.0483	0.0281
**15**	-212.9175879	-212.8387905	-212.7748368	0.0202	0.0220
**16**	-242.5331135	-242.3144903	-242.369516	0.0625	0.0315
**17**	-477.6171728	-477.4728309	-477.4895052	0.0272	0.0159
**18**	-529.2214381	-529.0224584	-529.1150429	0.0446	0.0262
**9-F**	-424.4663559	-424.2352136	-424.2902647	0.0379	0.0207

**15-F**	-411.2900394	-411.1859312	-411.1079566	0.0206	0.0206
**16-F**	-440.8234890	-440.5786931	-440.6515652	0.0520	0.0264
**17-F**	-675.9519587	-675.7967997	-675.8316555	0.0274	0.0160
**18-F**	-727.5154054	-727.3024828	-727.4153546	0.0396	0.0224
**9-SH$_3$**	-807.5667690	-807.4253664	-807.3761193	0.0360	0.0205
**15-SH$_3$**	-794.3742693	-794.2887776	-794.1970843	0.0149	0.0136
**16-SH$_3$**	-823.9901391	-823.7838053	-823.8314805	0.0628	0.0309
**17-SH$_3$**	-1059.054994	-1058.921308	-1058.927032	0.0223	0.0126
**18-SH$_3$**	-1110.669528	-1110.481130	-1110.562843	0.0445	0.0266

Table S19. The data points plotted in Figure 6, based on the data in Table S18.

	**Figure 6A**		**Figure 6B**	
**Compound**	***E*(^{3}n,π* - S$_0$) (eV)**	**MCI$_{tot}$(S$_0$ - ^{3}n,π*)**	***E*(^{3}π,π* - ^{3}n,π*) (eV)**	**MCI$_{tot}$(^{3}n,π*)**
**9**	4.27	0.0160	0.45	0.0229
**11**	4.33	0.0150	0.81	0.0228
**13**	4.47	0.0202	0.72	0.0281
**15**	2.14	-0.0018	1.74	0.0220
**16**	5.95	0.0310	-1.50	0.0315
**17**	3.93	0.0113	-0.45	0.0159
**18**	5.41	0.0184	-2.52	0.0262
**9-F**	6.29	0.0172	-1.50	0.0207
**15-F**	2.83	0.0000	2.12	0.0206
**16-F**	6.66	0.0256	-1.98	0.0264
**17-F**	4.22	0.0114	-0.95	0.0160
**18-F**	5.79	0.0171	-3.07	0.0224
**9-SH$_3$**	3.85	0.0155	1.34	0.0205
**15-SH$_3$**	2.33	0.0013	2.49	0.0136
**16-SH$_3$**	5.61	0.0319	-1.30	0.0309
**17-SH$_3$**	3.64	0.0098	-0.16	0.0126
**18-SH$_3$**	5.13	0.0179	-2.22	0.0266

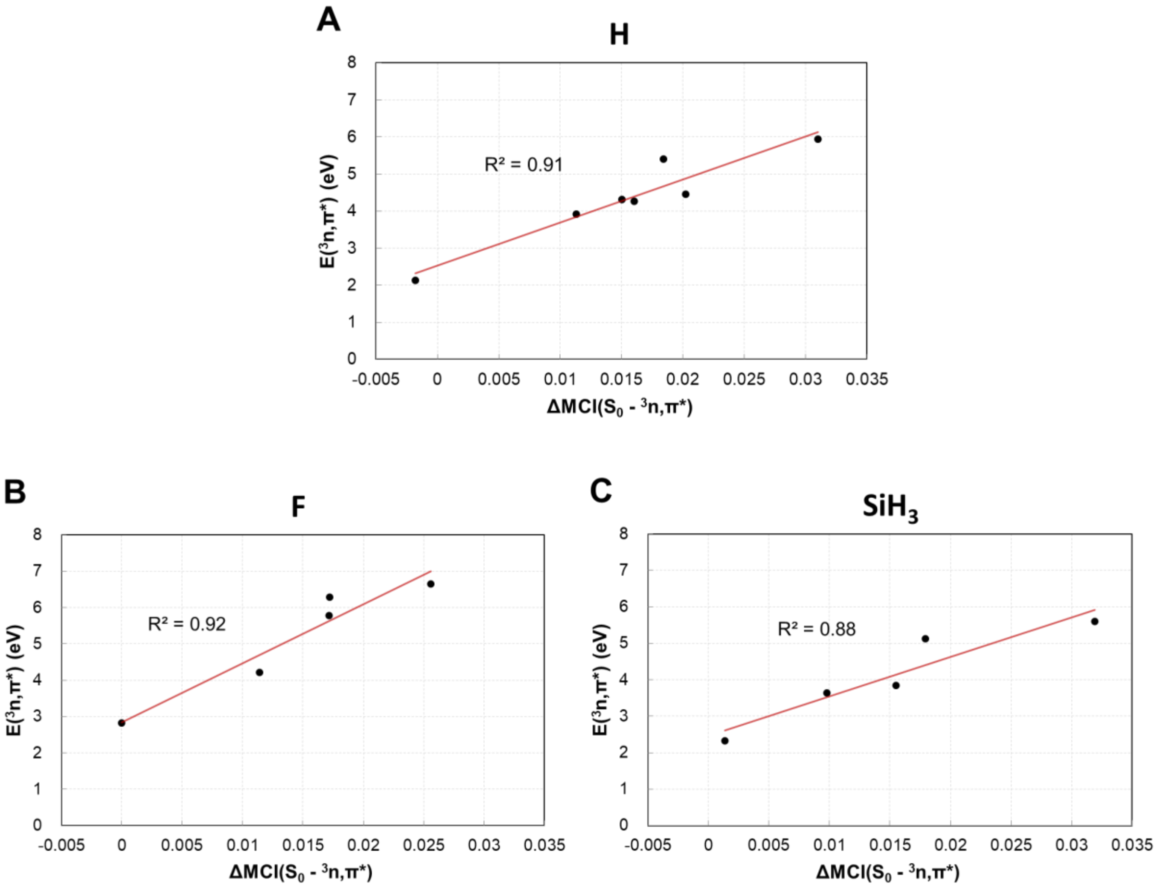

Figure S6. The plot of shown in Figure 6A, with the compounds grouped by their substitution (H, F or SiH_3, respectively).

4 Coordinates, total energies & number of imaginary frequencies

The optimized geometries of the systems presented in Figure 1 of the main manuscript are listed below. Input and output files for all calculations are available through the ioChem-BD repository[7] (DOI: 10.19061/iochem-bd-4-84) at https://iochem.udg.edu/browse/handle/100/6758.

1

S_0 state: Total energy = -248.213108124 Eh

Vertically excited $^3n\pi^*$ state: Total energy = -248.055016683 Eh

Number of imaginary frequencies in S_0 (NIMAG) = 0

C	0.00000	1.19177	-0.66852
C	0.00000	1.13720	0.71851
C	0.00000	-1.13720	0.71851
N	0.00000	0.00000	1.40924
H	0.00000	2.05162	1.30374
H	0.00000	2.14830	-1.17581
H	0.00000	-2.05162	1.30374
C	0.00000	-1.19177	-0.66852
H	0.00000	-2.14830	-1.17581
C	0.00000	0.00000	-1.37669
H	0.00000	0.00000	-2.46028

2

S_0 state: Total energy = -248.144214039 Eh,

Vertically excited $^3n\pi^*$ state: Total energy = -248.062563309 Eh

NIMAG (S_0) = 0

C	1.17371	0.61826	0.00000
C	1.14902	-0.74194	0.00000
C	-0.10923	-1.36952	0.00000
C	-1.25573	-0.61289	0.00000
C	-1.26632	0.80968	0.00000
H	2.08535	1.20221	0.00000
H	2.07069	-1.30710	0.00000
H	-0.16241	-2.45352	0.00000
H	-2.21634	-1.11590	0.00000
N	0.00000	1.30547	0.00000
H	0.07401	2.31454	0.00000

3

S_0 state: Total energy = -248.118841898 Eh

Vertically excited $^3n\pi^*$ state: Total energy = -248.061253497 Eh

NIMAG (S_0) = 0

C	-1.20078	0.63178	0.00000
C	-1.34908	-0.74762	0.00000
C	1.13903	-0.75586	0.00000
H	-2.04142	1.32210	0.00000
H	2.07301	-1.30549	0.00000
N	0.00000	1.27481	0.00000
H	0.01461	2.28765	0.00000
C	1.17202	0.61626	0.00000
H	2.07522	1.20977	0.00000
C	-0.10155	-1.40230	0.00000
H	-0.07934	-2.49128	0.00000

4

S_0 state: Total energy = -248.117454470 Eh

Vertically excited $^3n\pi^*$ state: Total energy = -248.073932135 Eh

NIMAG (S_0) = 0

C	0.00000	1.17844	0.60799
C	0.00000	1.17955	-0.76076
C	0.00000	0.00000	-1.55498
C	0.00000	-1.17955	-0.76076
H	0.00000	2.06859	1.22589
H	0.00000	2.15421	-1.24218
H	0.00000	-2.15421	-1.24218
N	0.00000	0.00000	1.27336
H	0.00000	0.00000	2.28218
C	0.00000	-1.17844	0.60799
H	0.00000	-2.06859	1.22589

5

S_0 state: Total energy = -234.872913989 Eh

Vertically excited $^3n\pi^*$ state: Total energy = -234.854583256 Eh

NIMAG (S_0) = 0

C	0.00000	1.20154	0.57087
C	0.00000	1.26855	-0.78028
C	0.00000	-1.26855	-0.78028
C	0.00000	-1.20154	0.57087
H	0.00000	2.06679	1.23802
H	0.00000	2.28089	-1.18222
H	0.00000	-2.28089	-1.18222
H	0.00000	-2.06679	1.23802
B	0.00000	0.00000	-1.70514

H	0.00000	0.00000	2.24439
N	0.00000	0.00000	1.24037

6

S_0 state: Total energy = -264.594758150 Eh

Vertically excited ^{3}nπ* state: Total energy = -264.485340555 Eh

NIMAG (S_0) = 0

C	0.00000	1.17314	-0.64101
C	0.00000	1.14237	0.74319
C	0.00000	-1.14237	0.74319
C	0.00000	-1.17314	-0.64101
H	0.00000	2.07949	-1.23109
H	0.00000	2.06518	1.31108
H	0.00000	-2.06518	1.31108
H	0.00000	-2.07949	-1.23109
N	0.00000	0.00000	1.41884
H	0.00000	0.00000	-2.30563
N	0.00000	0.00000	-1.28748

7

S_0 state: Total energy = -499.579948652 Eh

Vertically excited ^{3}nπ* state: Total energy = -499.497591192 Eh

NIMAG (S_0) = 0

Si	-0.55098	0.00021	-2.47234
C	-0.55098	1.37200	-1.21540
C	-0.55098	1.21328	0.12913
N	-0.55098	0.00087	0.75970
C	-0.55098	-1.21136	0.12931
C	-0.55098	-1.37017	-1.21535
H	-0.55098	2.40720	-1.54538
H	-0.55098	2.05101	0.82107
H	-0.55098	-2.04914	0.82119
H	-0.55098	-2.40547	-1.54507
H	-0.55098	0.00094	1.76672

8

S_0 state: Total energy = -551.225731344 Eh

Vertically excited ^{3}nπ* state: Total energy = -551.102860695 Eh

NIMAG (S_0) = 0

P	-0.55098	0.00049	-2.34214
C	-0.55098	1.32285	-1.21661
C	-0.55098	1.19818	0.14678
N	-0.55098	0.00071	0.76579
C	-0.55098	-1.19643	0.14684
C	-0.55098	-1.32082	-1.21668

H	-0.55098	2.32850	-1.62079
H	-0.55098	2.05863	0.80513
H	-0.55098	-2.05682	0.80524
H	-0.55098	-2.32654	-1.62076
H	-0.55098	0.00063	1.78077

9

S_0 state: Total energy = -226.130883063 Eh

Vertically excited $^3n\pi^*$ state: Total energy = -225.974037011 Eh

NIMAG (S_0) = 0

C	0.00000	0.00000	1.26708
C	0.00000	0.67317	-0.93332
C	0.00000	-0.67317	-0.93332
H	0.00000	1.37668	-1.74761
H	0.00000	-1.37668	-1.74761
N	0.00000	-1.04805	0.40274
H	0.00000	-1.99996	0.72709
N	0.00000	1.04805	0.40274
H	0.00000	1.99996	0.72709

10

S_0 state: Total energy = -226.097753170 Eh

Vertically excited $^3n\pi^*$ state: Total energy = -225.963475543 Eh

NIMAG (S_0) = 0

C	0.80954	-1.04102	0.00000
H	2.11482	0.76370	0.00000
H	-0.04231	2.09878	0.00000
H	-1.18775	-1.76321	0.00000
N	-0.59106	-0.95134	0.00000
C	1.14521	0.29428	0.00000
N	0.00000	1.09107	0.00000
C	-1.06396	0.29758	0.00000
H	-2.09208	0.61759	0.00000

11

S_0 state: Total energy = -242.171737246 Eh

Vertically excited $^3n\pi^*$ state: Total energy = -242.012623567 Eh

NIMAG (S_0) = 0

C	1.17042	0.44029	0.00000
H	1.31706	-1.64870	0.00000
C	-0.66313	-0.87831	0.00000
H	-1.26056	-1.77501	0.00000
H	-0.10753	2.09211	0.00000
N	0.00000	1.09188	0.00000
N	0.70968	-0.84673	0.00000

N	-1.13722	0.32052	0.00000

12

S_0 state: Total energy = -242.118574444 Eh

Vertically excited $^3n\pi^*$ state: Total energy = -241.995645427 Eh

NIMAG (S_0) = 0

C	0.74627	-1.05929	0.00000
H	2.09919	0.73797	0.00000
H	-0.09260	2.05592	0.00000
H	-1.29467	-1.63362	0.00000
N	-0.60997	-0.89136	0.00000
N	-1.09446	0.34251	0.00000
C	1.12359	0.28134	0.00000
N	0.00000	1.04992	0.00000

13

S_0 state: Total energy = -258.186515813 Eh

Vertically excited $^3n\pi^*$ state: Total energy = -258.022081720 Eh

NIMAG (S_0) = 0

C	0.00000	0.00000	1.23334
N	0.00000	0.62727	-0.95959
N	0.00000	-0.62727	-0.95959
H	0.00000	1.99978	0.56034
H	0.00000	-1.99978	0.56034
N	0.00000	1.01425	0.35097
N	0.00000	-1.01425	0.35097

14

S_0 state: Total energy = -258.153303977 Eh

Vertically excited $^3n\pi^*$ state: Total energy = -258.030746248 Eh

NIMAG (S_0) = 0

C	0.80113	-0.97001	0.00000
H	-0.03921	2.03163	0.00000
H	-1.22021	-1.67410	0.00000
N	-0.57553	-0.89812	0.00000
N	-1.07754	0.32401	0.00000
N	0.00000	1.02064	0.00000
N	1.14630	0.33383	0.00000

15

S_0 state: Total energy = -212.917587869 Eh

Vertically excited $^3n\pi^*$ state: Total energy = -212.838790522 Eh

NIMAG (S_0) = 0

C	0.00000	0.67564	-0.89730
C	0.00000	-0.67564	-0.89730

H	0.00000	1.34191	-1.74903
H	0.00000	-1.34191	-1.74903
B	0.00000	0.00000	1.39767
H	0.00000	2.08527	0.65377
H	0.00000	-2.08527	0.65377
N	0.00000	1.10910	0.42641
N	0.00000	-1.10910	0.42641

16

S_0 state: Total energy = -242.533113515 Eh

Vertically excited $^3n\pi^*$ state: Total energy = -242.314490324 Eh

NIMAG (S_0) = 0

H	0.00000	1.95316	0.77629
H	0.00000	-1.95316	0.77629
N	0.00000	1.02302	0.36598
N	0.00000	-1.02302	0.36598
C	0.00000	0.68208	-0.94657
H	0.00000	1.40350	-1.74679
C	0.00000	-0.68208	-0.94657
H	0.00000	-1.40350	-1.74679
N	0.00000	0.00000	1.16803

17

S_0 state: Total energy = -477.617172790 Eh

Vertically excited $^3n\pi^*$ state: Total energy = -477.472830862 Eh

NIMAG (S_0) = 0

C	0.00000	0.67297	-1.25463
C	0.00000	-0.67297	-1.25463
H	0.00000	1.31338	-2.12311
H	0.00000	-1.31338	-2.12311
N	0.00000	-1.19279	0.03325
H	0.00000	-2.19423	0.14610
N	0.00000	1.19279	0.03325
H	0.00000	2.19423	0.14610
Si	0.00000	0.00000	1.32458

18

S_0 state: Total energy = -529.221438054 Eh

Vertically excited $^3n\pi^*$ state: Total energy = -529.022458360 Eh

NIMAG (S_0) = 0

C	0.00000	0.67888	-1.25503
C	0.00000	-0.67888	-1.25503
H	0.00000	1.34469	-2.10342
H	0.00000	-1.34469	-2.10342
N	0.00000	-1.15509	0.02693

H	0.00000	-2.15253	0.21624
N	0.00000	1.15509	0.02693
H	0.00000	2.15253	0.21624
P	0.00000	0.00000	1.23052

19

S_0 state: Total energy = -115.318084583 Eh

Vertically excited $^3n\pi^*$ state: Total energy = -115.202520703 Eh

NIMAG (S_0) = 0

C	0.00000	0.65967	-0.32048
C	0.00000	-0.65967	-0.32048
C	0.00000	0.00000	0.92608
H	0.00000	1.59602	-0.85537
H	0.00000	-1.59602	-0.85537

20

S_0 state: Total energy = -102.099342938 Eh

Vertically excited $^3n\pi^*$ state: Total energy = -102.023873171 Eh

NIMAG (S_0) = 0

B	0.00000	0.00000	1.09688
C	0.00000	0.66357	-0.29025
C	0.00000	-0.66357	-0.29025
H	0.00000	1.48695	-1.00071
H	0.00000	-1.48695	-1.00071

21

S_0 state: Total energy = -131.703168943 Eh

Vertically excited $^3n\pi^*$ state: Total energy = -131.558501863 Eh

NIMAG (S_0) = 0

N	0.00000	0.00000	0.79997
C	0.00000	0.66974	-0.34446
C	0.00000	-0.66974	-0.34446
H	0.00000	1.68336	-0.73317
H	0.00000	-1.68336	-0.73317

22

S_0 state: Total energy = -366.800005862 Eh

Vertically excited $^3n\pi^*$ state: Total energy = -366.683761031 Eh

NIMAG (S_0) = 0

C	0.00000	0.66817	-0.79194
C	0.00000	-0.66817	-0.79194
Si	0.00000	0.00000	0.90090
H	0.00000	1.43708	-1.55467
H	0.00000	-1.43708	-1.55467

23

S_0 state: Total energy = -418.381720751 Eh

Vertically excited $^3n\pi^*$ state: Total energy = -418.245121531 Eh

NIMAG (S_0) = 0

C	0.00000	0.67035	-0.77426
C	0.00000	-0.67035	-0.77426
H	0.00000	1.54242	-1.41602
H	0.00000	-1.54242	-1.41602
P	0.00000	0.00000	0.80821

References

1 Havenith, R. W. A.; De Proft, F.; Fowler, P. W.; Geerlings, P. Σ-Aromaticity in H_3^+ and Li_3^+: Insights from Ring-Current Maps. *Chem. Phys. Lett.* **2005**, *407*, 391-396.

2 Sadjadi, S. On the Topology of the Electron Density of H_3^+. *Struct. Chem.* **2017**, *28*, 1445-1452.

3 Foroutan-Nejad, C.; Rashidi-Ranjbar, P., Chemical Bonding in the Lightest Tri-Atomic Clusters; H_3^+, Li_3^+ and B_3^-. *J. Mol. Struct. THEOCHEM* **2009**, *901*, 243-248.

4 Wu, W.; Ma, B.; Wu, J. I-C.; Schleyer, P. v. R.; Mo, Y., Is Cyclopropane Really the s-Aromatic Paradigm? *Chem. Eur. J.*, **2009**, *15*, 9730 – 9736.

5 Proos Vedin, N.; Escayola, S.; Radenković, S.; Solà, M.; Ottosson, H., The n,π* States of Heteroaromatics: When are They the Lowest Excited States and in What Way Can They Be Aromatic or Antiaromatic? *J. Phys. Chem. A* **2024**, *128*, 4493–4506.

6 Schuurman, M. S.; Giegerich, J.; Pachner, K.; Lang, D.; Kiendl, B.; MacDonell, R. J.; Kruger, A.; Fischer, I., Photodissociation Dynamics of Cyclopropenylidene, *c*-C_3H_2, *Chem. Eur. J.* **2015**, *21*, 14486 – 14495.

7 Álvarez-Moreno, M.; de Graaf, C.; López, N.; Maseras, F.; Poblet, J. M.; Bo, C., *J. Chem. Inf. Model.* **2015**, 55, 1, 95–103.
